# Non-aqueous selective synthesis of orthosilicic acid and its oligomers

**DOI:** 10.1038/s41467-017-00168-5

**Published:** 2017-07-26

**Authors:** Masayasu Igarashi, Tomohiro Matsumoto, Fujio Yagihashi, Hiroshi Yamashita, Takashi Ohhara, Takayasu Hanashima, Akiko Nakao, Taketo Moyoshi, Kazuhiko Sato, Shigeru Shimada

**Affiliations:** 1 Interdisciplinary Research Center for Catalytic Chemistry, National Institute of Advanced Industrial Science and Technology (AIST), Tsukuba Central 5, 1-1-1 Higashi, Tsukuba, 305-8565 Japan; 2 Neutron Science Section, J-PARC Center, Japan Atomic Energy Agency, Shirakata-shirane 2-4, Tokai, 319-1195 Japan; 3 Neutron Science and Technology Center, Comprehensive Research Organization for Science and Society, IQBRC Building, Shirakata 162-1, Tokai, 319-1106 Japan

## Abstract

Orthosilicic acid (Si(OH)_4_) and its small condensation compounds are among the most important silicon compounds but have never been isolated, due to their instability. These compounds would be highly useful building blocks for advanced materials if they became available at high purity. Here we show a simple procedure to selectively synthesize orthosilicic acid and its dimer, cyclic trimer and tetramer in organic solvents. Isolation of orthosilicic acid, the dimer and the cyclic tetramer as hydrogen-bonded crystals with tetrabutylammonium halides and the cyclic trimer as solvent-containing crystals is also described. The solid-state structures of these compounds are unambiguously clarified by single crystal X-ray and neutron diffraction studies. The usefulness of orthosilicic acid and its oligomers prepared by the new procedure is demonstrated by the synthesis of functionalized oligosiloxanes.

## Abstract (web summary)

Orthosilicic acid is essential to many natural and synthetic materials but notoriously difficult to isolate, limiting its use in materials synthesis. Here, the authors successfully synthesize and stabilize orthosilicic acid and its oligomers, making available a new family of building blocks for silicon oxide-based materials.

## Introduction

**Fig. 1:**
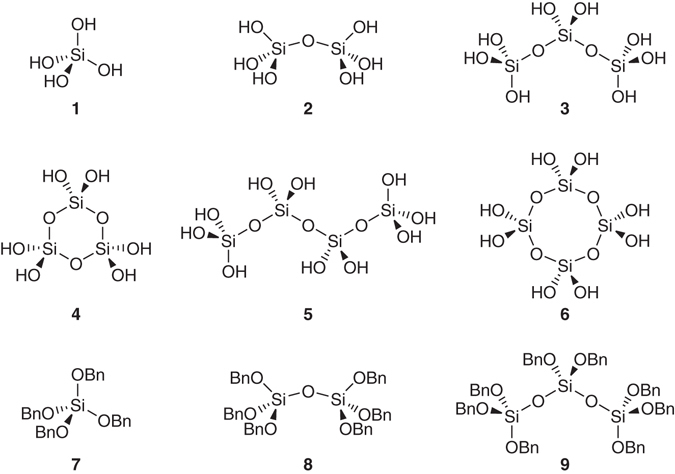
Chemical structures of compounds. Orthosilicic acid (**1**), its condensation oligomers (**2**: disilicic acid, **3** and **4**: linear and cyclic trisiloxanes, **5** and **6**: linear and cyclic tetrasiloxanes), and starting materials **7**–**9** for the synthesis of **1**-**6** (Bn = CH_2_C_6_H_5_, benzyl)

Orthosilicic acid is one of the most fundamental silicon compounds. It is the smallest building block for silica and silicates, the most abundant natural substances comprising the earth’s crust^1^. A huge amount of orthosilicic acid (10^17^ mol, ~ 10^13^ tonnes) naturally exists in seas and rivers and plays a key role in the continental cycle of silicon by biotic and abiotic processes^2–4^, though its average concentration is low (70 μM in seawater)^2^. Orthosilicic acid is also essential in the development and cellular metabolism of gramineous plants^5, 6^ and the development of bone, connective tissue, skin, hair, and nails in humans and other animals^7–9^. Furthermore, orthosilicic acid and its small condensation compounds, such as its dimer (disilicic acid), linear and cyclic trimers (trisiloxanes), and tetramers (tetrasiloxanes) (Fig. 1), are key intermediates in the synthesis of advanced silicon oxide-based materials, such as mesoporous silica, zeolites, and silicones^10–14^.

Due to the importance of orthosilicic acid, its preparation and condensation reaction have long been studied^1^. In 1820, Berzelius reported the preparation of “soluble silica” by reacting ammonium hydroxide with hydrofluorosilicic acid^15^. As summarized by Hurd in 1938, a number of studies were done on silicic acid gel formations during the 19th and early 20th centuries^16^. Broader and deeper studies on orthosilicic acid, its condensation reaction, intermediate oligomers, and polymers have been continued to date^1, 17–19^. Various spectroscopic and chemical methods have been used for the analysis of orthosilicic acid and its condensation reaction. ^29^Si NMR spectroscopy is the most reliable method for the identification of various intermediate species during the condensation reaction. This method has identified the existence of various linear, branched, and cyclic oligomeric species (anionic and/or neutral forms) including compounds **1**–**6** in Fig. 1 ^20–22^.

Orthosilicic acid is usually generated by the hydrolysis of tetraalkoxysilanes or silicon tetrachloride^11^. Although a wide variety of materials have been synthesized through this hydrolytic method, its usage severely limits applicable synthetic methodologies. Under the hydrolytic conditions, the hydrolysis and the condensation reactions of the resulting species occur concurrently, generating various intermediate species unselectively; thus, orthosilicic acid and its small condensation compounds have never been isolated^1^. Precise step-by-step synthesis of well-defined structures similar to organic synthesis is impossible by the conventional hydrolytic method.

Therefore, the synthesis of orthosilicic acid and its small condensation oligomers at high purity is highly desirable, particularly from the point of view of materials synthesis. The availability of these species at high purity as starting materials in organic solvents or in isolated forms can completely alter and considerably broaden synthetic methodologies for materials synthesis, leading to the development of stepwise and highly selective transformations for the precise control of material structures, and contributes to the creation of new fields in material science of silicas, silicates, zeolites, silicones, and other silicon oxide-based materials. Such selective transformations have well established in organic synthesis and applied to the design and synthesis of organic materials. Furthermore, these species have a potential to alter materials processing by creating new wet processes for making silicon oxide layers on flexible plastic substrates as well as on silicon wafers and for epitaxial growth of silicon dioxide on a crystal plane of metal or semiconductor at ordinary temperature and pressure.

In this report, we present the isolation of orthosilicic acid and its small condensation oligomers as hydrogen-bonded crystals with or without ammonium halides by developing a non-hydrolytic synthetic method for these highly sensitive compounds. Their molecular structures are precisely revealed by single crystal X-ray and neutron diffraction studies. Furthermore, usefulness of the new synthetic method is demonstrated by converting orthosilicic acid and its oligomers to functionalized oligosiloxanes.

## Results

### Synthesis of orthosilicic acid

As mentioned above, orthosilicic acid (**1**) is usually generated under hydrolytic conditions in aqueous solution. Under such conditions, **1** can stably exist without condensation only if the concentration is very low. Therefore, we decided to use a completely different reaction and conditions in order to stably generate a relatively high concentration of **1** for its isolation. We selected a non-aqueous hydrogenolysis reaction of benzyloxysilanes, which is similar to the hydrogenolysis of benzyl ethers commonly used in organic synthesis for the protection/de-protection of alcohol functionality^23^.

The hydrogenolysis reaction of tetrakis(benzyloxy)silane (Si(OCH_2_C_6_H_5_)_4_, **7**) occurred completely under the conditions suitable for organic silanol synthesis, which we have previously developed^24^: 1 atm of H_2_ in THF with a Pd/C catalyst. However, ^29^Si NMR analysis of the reaction mixture revealed no signal corresponding to Si(OH)_4_ **1** at all, suggesting further condensation reaction of **1** occurred, resulting in oligomers and/or polymers. Despite the result, we expected that this procedure still had potential as a synthetic method of orthosilicic acid because many factors remained to be tuned. It is thought that a high concentration of soluble silicon (orthosilicic acid and/or its small condensation oligomers) is stabilized in some plants, such as diatoms, which take up orthosilicic acid as a silicon source for their uniquely shaped cell walls^25^. Biomolecules are probably involved in the stabilization via hydrogen bonds and/or electrostatic interactions^25^, and the stabilizing effects of various compounds have been examined^26, 27^. A recent report demonstrated that simple polyethylene glycols also have a stabilizing effect, in which hydrogen bonds are thought to be the key factor^26^.

Next, we tested various solvents, including those with the ability to form hydrogen bonds with **1** (Supplementary Table 1). Although most conventional solvents, including hexane, toluene, ethyl acetate, CH_2_Cl_2_, CH_3_CN, and dimethyl sulfoxide (DMSO), did not show a positive effect, some amide-type solvents considerably suppressed the condensation reaction. When the reaction was carried out in *N,N*-dimethylacetamide (DMAc), a small amount (13%) of **1** was obtained in addition to a 34% yield of **2** (Supplementary Table 1, Entry 10). Using mixed solvents attained further improvement; *N*-methylacetamide (MMAc)-DMAc mixed solvent (90:10 volume ratio) afforded **1** in 85% yield (Supplementary Table 1, Entry 12).

A solution treated with Pd/Cs under H_2_ becomes acidic due to the residual acids and/or PdCl_2_ in Pd/Cs depending on the suppliers^28^. Because the acid formation will accelerate the condensation reaction, further improvement of the yield of **1** may be possible by adding a suitable base to adjust the pH of the reaction system. Actually, a weak base, aniline, was proved to be effective. Representative results are summarized in Supplementary Table 2. After carefully tuning the amounts of Pd/C catalyst and aniline in a DMAc solvent, the yield of **1** increased up to 90% with a small amount of **2** (Supplementary Table 2, Entry 7). Furthermore, we finally succeeded in obtaining **1** in nearly quantitative yield in the MMAc-DMAc mixed solvent with a suitable amount of aniline (96%, Supplementary Table 2, Entry 10)^29^. We have confirmed that decomposition of the solvents did not happen at least for DMAc at a detectable level by ^1^H NMR, which forms an amine and is known often to happen for some amide-type solvents such as DMF.

### Synthesis of disilicic acid, cyclic trimer 4 and tetramer 6

As described above, **2** was formed as a by-product of the hydrogenolysis of **7** due to the condensation of **1**. However, it was difficult to obtain **2** selectively by the condensation of **1**. Therefore, we applied the hydrogenolysis procedure for the selective synthesis of **2** from hexakis(benzyloxy)disiloxane (**8**), which was easily synthesized from commercially available hexachlorodisiloxane. Suitable reaction conditions for **2** were found based on the reaction conditions for the synthesis of **1**. The hydrogenolysis of **8** in 1,1,3,3-tetramethylurea (TMU) and DMAc with Pd/C catalyst in the presence of a suitable amount of aniline under hydrogen atmosphere (1 atm) afforded **2** in yields of 95% and 94%, respectively. A similar reaction in MMAc-DMAc mixed solvent (90/10) did not require aniline and afforded **2** in a yield of 96%.

We applied the same procedure for the synthesis of linear trisiloxane **3** from octakis(benzyloxy)trisiloxane (**9**), which was easily synthesized from commercially available octachlorotrisiloxane. However, the maximum yield of **3** remained 47% when the reaction of **9** was performed in DMAc with Pd/C catalyst and aniline (aniline/Pd = 1.49) under hydrogen atmosphere (1 atm). However, from the hydrogenolysis of **9** we found the formation of considerable amounts of cyclotrisiloxane **4** in most cases.

Next, we turned our attention to the selective synthesis of **4** because cyclic structures are commonly observed in various silicon oxide materials, such as silicates and zeolites, and cyclic siloxanes such as **4** and **6** would be highly useful building blocks for advanced material synthesis. After carefully tuning the reaction conditions, we found that the amount of aniline considerably affected the cyclization, and a 94% yield was achieved for **4** when the hydrogenolysis reaction of **9** was performed with Pd/C catalyst and aniline (aniline/Pd = 0.75) in DMAc under hydrogen atmosphere (1 atm).

Although tetrasiloxanes **5** and **6** could not be selectively synthesized, we succeeded in isolating **6** as co-crystals with tetrabutylammonium chloride. When the hydrogenolysis reaction of **8** was performed in TMU in the absence of aniline, ^29^Si NMR analysis showed the formation of **2** (40%), **4** (15%), and **6** (17%) as the main products. Attempted crystallization of a product from this mixture by the addition of tetrabutylammonium chloride (vide infra) afforded single crystals containing **6**, ^*n*^Bu_4_NCl, and TMU ([**6**·4(^*n*^Bu_4_NCl)·2TMU], 42%). Based on this yield, the equilibrium between **2**, **4**, **6**, and other species shifted to the formation of **6** by the preferred crystallization of [**6**·4(^*n*^Bu_4_NCl)·2TMU].

### Characterization of orthosilicic acid and its oligomers

The structures of **1**, **2**, **4**, and **6** were unambiguously characterized by ^1^H and ^29^Si NMR, infrared (IR) and high resolution mass spectroscopies, and single crystal X-ray analysis. Neutron diffraction analysis was also performed for **1**. The ^29^Si NMR spectrum of the reaction mixture obtained by the hydrogenolysis of **7** in a MMAc/DMAc mixed solvent (Supplementary Table 2, Entry 10) showed a sharp intense signal for **1** at −72.2 parts per million (ppm) with a tiny signal for **2** at −80.8 ppm (Supplementary Fig. 2b)^30, 31^. The ^1^H NMR spectrum of the sample obtained in DMAc (Supplementary Table 2, Entry 7) showed a signal for the four hydrogen atoms of **1** at 5.69 ppm and the six hydrogen atoms of **2** at 5.80 ppm (Supplementary Fig. 2a). A two-dimensional ^1^H–^29^Si NMR analysis confirmed the correlations of the respective ^1^H and ^29^Si signals for **1** and **2** (Supplementary Fig. 2c). Further evidence of **1** was obtained by the high-resolution time-of-flight mass spectroscopy (TOF-MS); analysis of the reaction mixture in CH_3_CN in negative ion mode clearly showed a signal at m/z 130.9568, corresponding to the Cl-bound anion, [Si(OH)_4_Cl]^–^ (calculated for H_4_O_4_SiCl^−^: 130.9573, Supplementary Fig. 3a, b). Deuterium analogue Si(OD)_4_ **1**-*d* was also synthesized by performing the reaction using D_2_ instead of H_2_. The IR spectra of crystals of the hydrogen-bonded complex of **1** and **1**-*d* with tetrabutylammonium chloride (vide infra) clearly showed the OH and OD stretching bands at different positions (Supplementary Fig. 3c).

Furthermore, ^1^H, ^29^Si, and two-dimensional ^1^H–^29^Si NMR spectra of **2** obtained in DMAc (Supplementary Fig. 4) and crystalline **4** containing TMU (vide infra, Supplementary Fig. 5) showed very clear signals for these species^30, 31^. High-resolution TOF-MS spectra of these species in negative ion mode also clearly confirmed the masses of the corresponding Cl-bound anion species. Dissolution of the crystal [**6**·4(^*n*^Bu_4_NCl)·2TMU] in DMF-d_7_/THF-d_8_ mixed solvent clearly showed ^1^H, ^29^Si and ^1^H-^29^Si correlation NMR signals for **6** (Supplementary Fig. 6)^31, 32^.

**Fig. 2:**
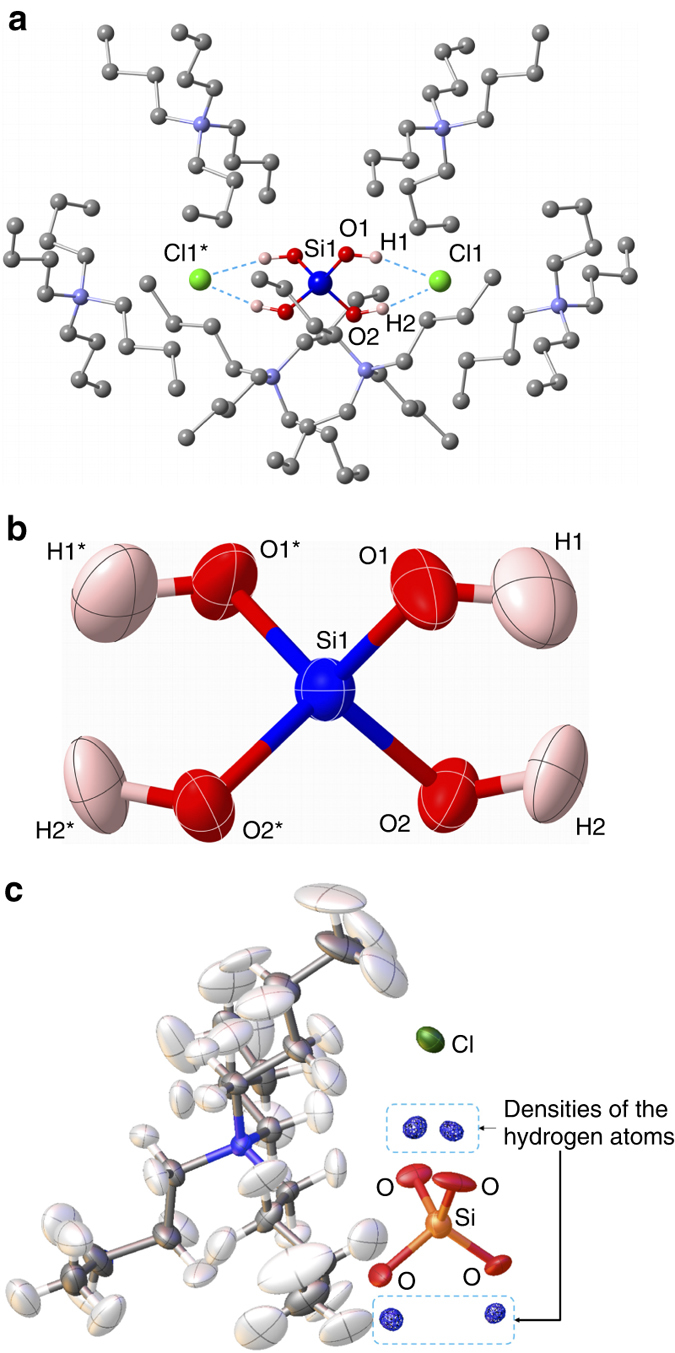
Structure of orthosilicic acid in the [Si(OH)_4_·2(^*n*^Bu_4_NCl)] crystal. **a** Arrangement of Si(OH)_4_ in the crystal determined by neutron diffraction. The hydrogen-bonded [Si(OH)_4_·2Cl^–^] moiety is surrounded by [^*n*^Bu_4_N^+^]. Hydrogen atoms in the ^*n*^Bu groups were omitted for clarity. **b** A thermal ellipsoid plot (50% probability level) of Si(OH)_4_ in [Si(OH)_4_·2(^*n*^Bu_4_NCl)] determined by neutron diffraction. **c** D-Fourier map with the neutron diffraction data for [Si(OH)_4_·2(^*n*^Bu_4_NCl)] and a structural model without the H atoms of the Si(OH)_4_ moiety. Residual neutron scattering length density derived from the four hydrogen atoms of the Si(OH)_4_ moiety is clearly observed. Measurement details are provided in Supplementary Methods

As we succeeded in obtaining a relatively high concentration (up to 90 mM) of **1** in organic solvents at high purity, crystallization of **1** was attempted. Initial attempts to obtain a single crystal of pure **1** were unsuccessful. Because organic silanols form hydrogen-bonded complex crystals with various donor molecules, such as amines and ammonium salts, we examined various additives to obtain a single crystal of the hydrogen-bonded complex of **1** ^33, 34^. Although the addition of amine additives resulted in the condensation of **1**, single crystals of hydrogen-bonded complexes of **1**, in which neutral forms of **1** are stabilized by hydrogen bonds, were successfully obtained by the addition of tetrabutylammonium halides (^*n*^Bu_4_NX, where ^*n*^Bu = CH_3_(CH_2_)_3_ and X = Cl or Br). Single crystals of [**1**·2(^*n*^Bu_4_NCl)] and [**1**·2(^*n*^Bu_4_NBr)] were grown from a 1:2 mixture of **1** and ^*n*^Bu_4_NX in TMU-diethyl ether. The solid-state structure of [**1**·2(^*n*^Bu_4_NCl)] was determined by single crystal X-ray and neutron diffraction studies. As shown in Fig. 2a, the Si(OH)_4_ molecule forms hydrogen bonds with two Cl^–^ anions and is isolated from other Si(OH)_4_ molecules by the surrounding ^*n*^Bu_4_N^+^ cations. The neutron diffraction study unambiguously determined the positions of hydrogen atoms, clearly showing a neutral Si(OH)_4_ structure (Fig. 2b,c) in which the SiO_4_ moiety is in an almost ideal tetrahedral structure. The Si-O and O-H distances (Table 1) determined by this study agree with the values obtained by theoretical calculations^35, 36^.

**Table 1:** Selected bond distances [Å] and angles [°] of Si(OH)_4_ in the crystal determined by neutron and X-ray diffraction

	Neutron	X-ray
Si1-O1	1.610 (4)	1.6186 (11)
Si1-O2	1.621 (4)	1.6258 (9)
O1-H1	0.946 (7)	0.84
O2-H2	0.950 (6)	0.84
O1-Si1-O1*	113.3 (3)	112.36 (6)
O1-Si1-O2	107.39 (17)	108.21 (5)
O1-Si1-O2*	109.95 (17)	109.55 (5)
O2-Si1-O2*	108.8 (3)	108.91 (5)

**Fig. 3:**
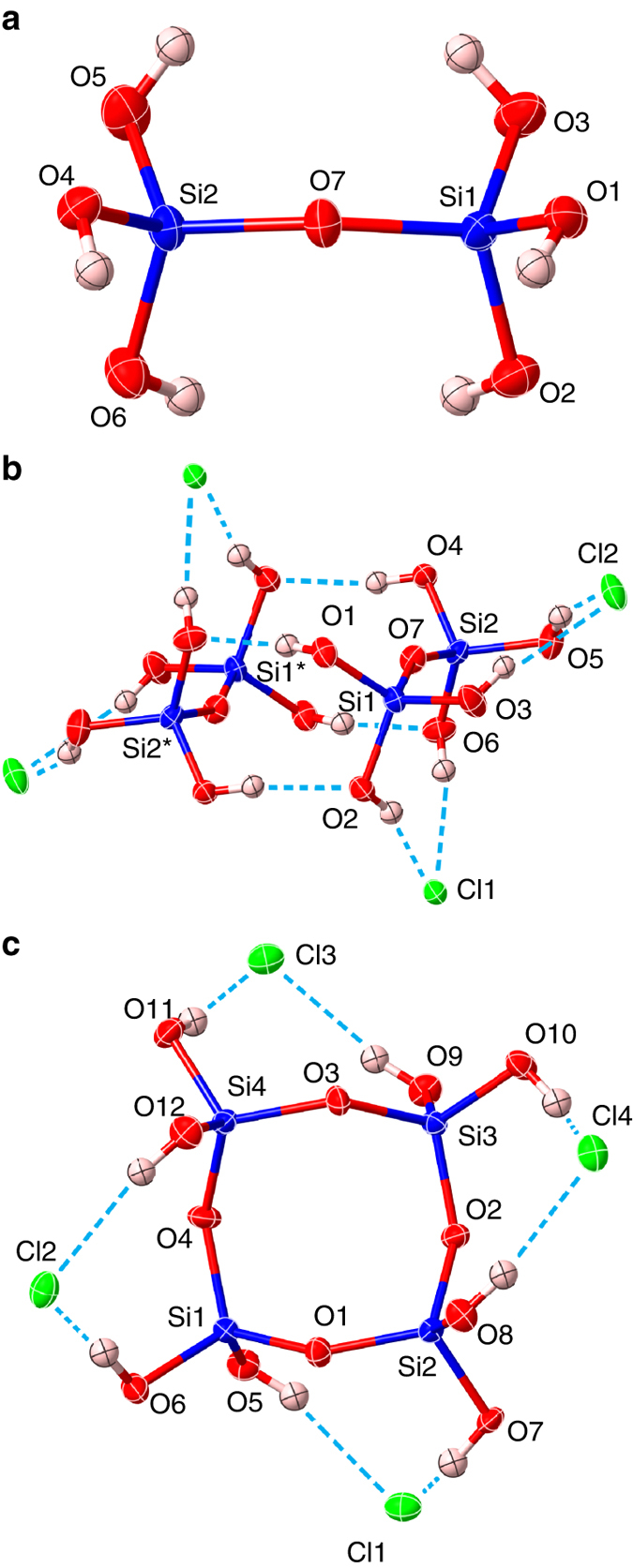
Structures of disilicic acid 2 and cyclotetrasiloxane 6. **a** A thermal ellipsoid plot (50% probability level) of (HO)_3_SiOSi(OH)_3_ in [**2**·2(^*n*^Bu_4_NCl)] determined by X-ray diffraction. **b** Hydrogen-bonded arrangement of two (HO)_3_SiOSi(OH)_3_ molecules and four Cl^−^ anions in the crystal determined by X-ray diffraction. **c** A thermal ellipsoid plot (50% probability level) of **6** with four Cl^−^ anions in [**6**·4(^*n*^Bu_4_NCl)·2TMU] determined by X-ray diffraction

Crystallization of **2** was achieved similarly by the addition of ^*n*^Bu_4_NCl. Compound **6** was also crystallized from a mixture of **2**, **4**, and **6** using ^*n*^Bu_4_NCl (vide supra). Figure 3a shows the structure of **2** in the crystal; two molecules of **2** form a dimeric structure through four hydrogen bonds with further hydrogen-bonding to chlorine anions (Fig. 3b). Compound **6** forms a hydrogen-bonded structure in the crystal, in which every two SiOH moieties are connected to one chlorine anion through hydrogen bonds (Fig. 3c).

**Fig. 4:**
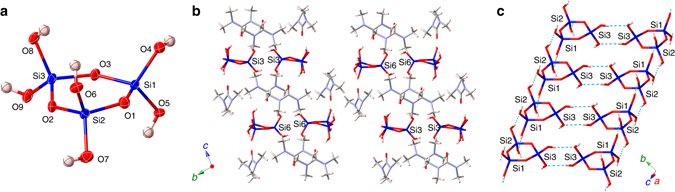
Structure of cyclotrisiloxane 4 determined by single crystal X-ray diffraction. **a** A thermal ellipsoid plot (50% probability level) of **4** in [**4**·2TMU] determined by X-ray diffraction. **b** A view along the *a* axis. **c** A view showing the columnar arrangement of **4** through hydrogen bonds in the crystal along the *a* axis

On the other hand, we succeeded in obtaining single crystals of **4** without the addition of ^*n*^Bu_4_NCl from the reaction mixture by using TMU as a solvent instead of DMAc (yield of **4** was ~ 95%). The crystal contained TMU as a co-crystallization solvent. Figure 4a shows the molecular structure of one of two crystallographically independent molecules of **4**. The six-membered ring of **4** is rather planar, but not in a chair or boat conformation, which is in good agreement with the structure suggested by recent theoretical calculations^37^. In the crystal, each of the two crystallographically independent molecules of **4** forms infinite columns through hydrogen bonds along the *a*-axis (Fig. 4b). Figure 4c shows a part of one of the indefinite columns, in which each **4** molecule connected to three adjacent **4** molecules through hydrogen bonds. Each **4** molecule is further connected to two TMU molecules through hydrogen bonds on both sides of the six-membered ring.

### Stability of orthosilicic acid and its oligomers in solution

**Fig. 5:**
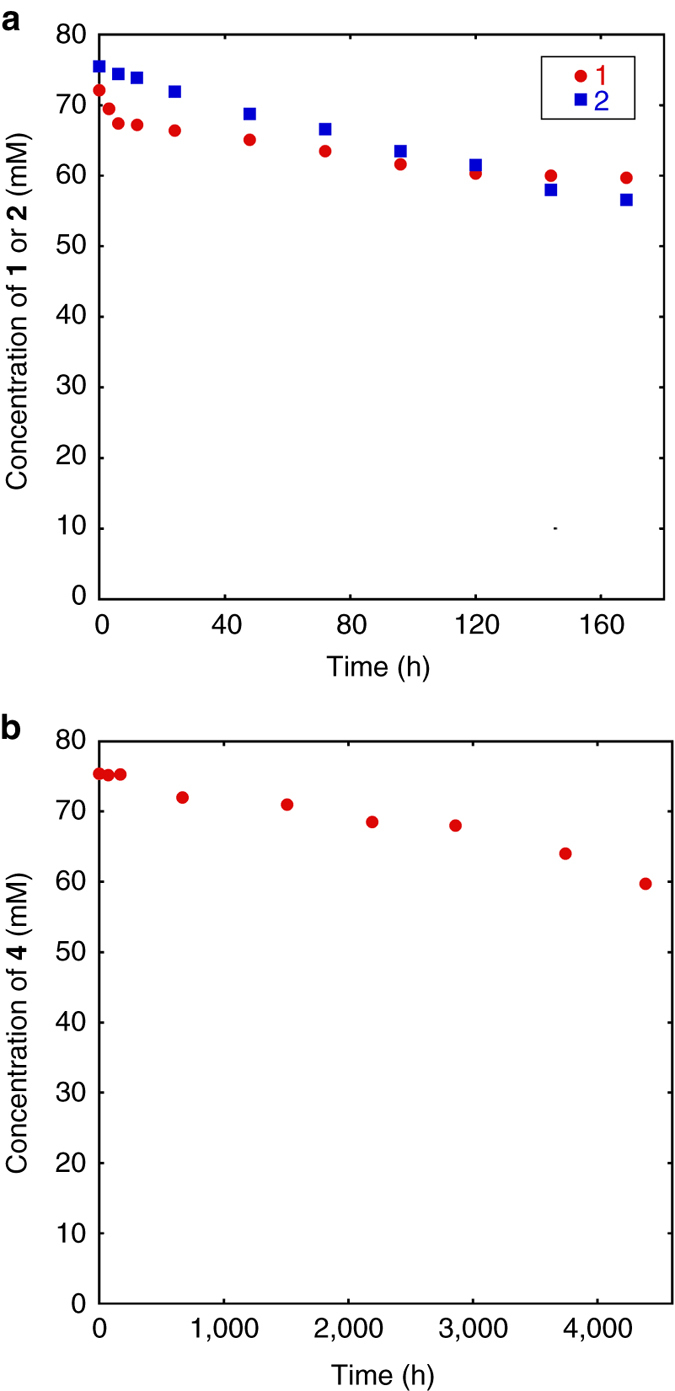
Time-dependent concentration changes in 1 and its oligomers. **a** The time-dependent concentration changes in **1** and **2**. Each sample of **1** and **2** was prepared separately by the hydrogenolysis reaction of **7** and **8**, respectively, in DMAc, subsequent filtration of the catalyst, and dilution with a small amount of deuterated-THF to obtain the starting sample containing **1** (72 mM) and **2** (76 mM). Each sample was periodically (up to 168 h) monitored by ^29^Si NMR spectroscopy using 1,4-bis(trimethylsilyl)benzene as an internal standard. **b** The time-dependent concentration changes in **4**. The initial sample of **4** was prepared similarly as above from **9** in DMAc. The change in concentration of **4** (initial concentration 75 mM) was periodically (up to ~ 4400 h, 6 months) monitored by ^29^Si NMR spectroscopy

Compound **1** stably exists in an aqueous solution only at very low concentrations^1^ and readily condenses to form oligomers and polymers. The stability of **2** is expected to be similar to that of **1**. However, under our conditions **1** and **2** can exist for a much longer period of time, even at room temperature (~ 22 °C), at a relatively high concentration of up to 90 mM in the case of **1**. Figure 5a shows the time-dependent concentration change in **1** and **2** respectively prepared in DMAc under the optimized conditions. The initial concentrations of **1** and **2** in the starting samples were 72 mM and 76 mM, respectively. The condensation reaction of **1** and **2** occurred very slowly under these conditions and the concentrations of **1** and **2** after 1 week (168 h) at room temperature were 60 mM and 57 mM, corresponding to 83% and 75% of the initial concentrations, respectively.

On the other hand, the stability of **4** was very surprising. Cyclotrisiloxane rings have a higher ring-strain and are less stable than larger ring cyclosiloxanes^38, 39^. Compound **4** prepared in DMAc (75 mM) exhibited almost no change after 168 h at 22 ºC. Therefore, we monitored its stability for 6 months. As shown in Fig. 5b, the concentration of **4** decreased very slowly and remained at 60 mM (80% of the initial concentration) after 6 months.

### Synthesis of orthosilicic acid by hydrolysis in DMAc

Based on the above observations, we anticipated that the hydrolysis of tetraalkoxysilanes in TMU or DMAc as a solvent under appropriate conditions would provide an easier and more practical method for the preparation of **1**. As expected, hydrolysis of tetraethoxysilane Si(OCH_2_CH_3_)_4_ in DMAc with a slight excess of water in the presence of 1 mol% HCl for 30 min at room temperature and subsequent neutralization with triethylamine afforded **1** almost quantitatively (concentration: 0.13 M). Higher concentrations of **1** up to 0.44 M were more easily obtained by the hydrolysis of tetramethoxysilane Si(OCH_3_)_4_ in DMAc under similar reaction conditions. A ^29^Si NMR spectrum of the reaction mixture clearly showed the formation of **1** in high selectivity (Supplementary Fig. 7).

### Synthesis of functionalized oligosiloxanes

**Fig. 6:**
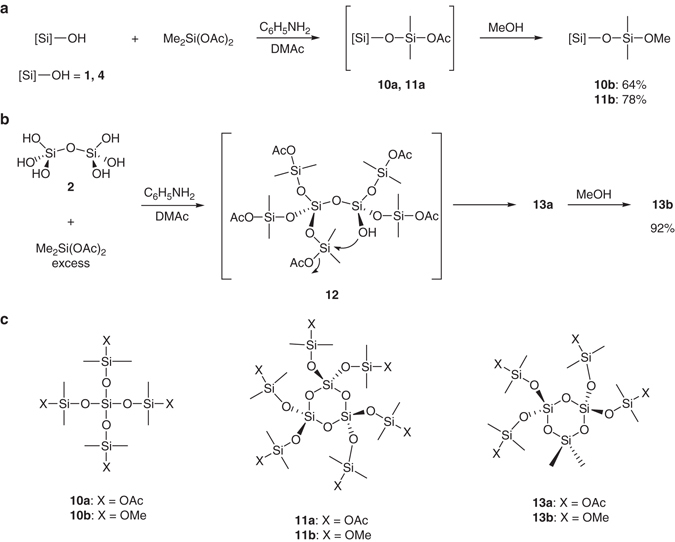
Synthesis of functionalized oligosiloxanes. **a** Reaction scheme for the synthesis of **10** and **11** respectively from **1** and **4**. **b** Reaction scheme for the synthesis of **13** from **2**. **c** Chemical structures of **10**, **11**, and **13**

In order to demonstrate the usefulness of our procedure, functionalized oligosiloxanes were prepared from **1**, **2**, and **4** (Fig. 6). A DMAc solution of **1** or **4** prepared by the hydrogenolysis reaction of **7** or **9** was treated with an excess of Me_2_Si(OAc)_2_ in the presence of aniline converting all Si-OH groups to Si-O-SiMe_2_(OAc) groups with good selectivity to give **10a** or **11a** (Fig. 6a). On the other hand, a similar reaction of **2** prepared from **8** with Me_2_Si(OAc)_2_ unexpectedly afforded cyclotrisiloxane **13a** with high selectivity, which was probably formed by the intramolecular cyclization of the intermediate **12** (Fig. 6b). Compounds **10a**, **11a** and **13a** were further converted to more stable methoxy compounds **10b**, **11b** and **13b**, respectively, by the treatment with methanol and their structures were characterized by ^1^H, ^13^C, ^29^Si, and ^1^H-^29^Si 2D NMR spectroscopies and high-resolution mass spectroscopy (Supplementary Figs. 8–21). Compounds **10a** and **10b** were obtained together with small amounts of **13a** and **13b**, respectively. The synthesis of compounds **10**, **11**, and **13** from **1**, **2**, and **4**, respectively, is only possible in the absence of water because Me_2_Si(OAc)_2_ readily reacts with water. Although the synthesis of **10b** in a low yield by an alternative method is known^40^, compounds **11** and **13** are difficult to synthesize by known procedures. The functionalized oligosiloxanes **10**, **11**, and **13** can be used as components of new silicone materials by the transformation of the functional groups. Furthermore, ring-opening polymerization of cyclotrisiloxanes **11** and **13** would readily occur to provide functionalized polysiloxanes^41^.

In summary, we have developed a new synthetic procedure for orthosilicic acid **1**, disilicic acid **2**, cyclotrisiloxane **4**, and cyclotetrasiloxane **6** based on the hydrogenolysis of benzyloxysilanes in organic solvents and achieved the crystallization of these species. The key to the stabilization of these highly reactive compounds is the use of suitable amide or urea solvents and controlling the acidity of the reaction media. Furthermore, based on the results, an easier and more practical procedure for synthesizing orthosilicic acid by the hydrolysis of alkoxysilanes was also developed. This procedure readily provides a high concentration of orthosilicic acid at high purity. The current findings make the family of silicic acid compounds available as building blocks for materials synthesis and processing, as demonstrated by the synthesis of functionalized oligosiloxanes, which would lead to new well-defined polysiloxanes. The results will alter the synthetic and processing methodologies of inorganic and organometallic silicon oxide-based materials and lead to a wide variety of advanced materials with well-defined structures, opening up a new field in materials science.

## Methods

### General information

Quantification of the products was performed by ^29^Si NMR spectroscopy, for which appropriate pulse delay time was determined as described in (Supplementary Fig. 1 and Supplementary Table 3). Total yields of orthosilicic acid and its oligomers determined by the ^29^Si NMR spectroscopy were compared with those obtained by the conventional molybdenum yellow method, showing reasonably good agreement (Supplementary Table  4). Details of instrumentation, materials, and synthetic procedures are shown in Supplementary Methods. See Supplementary Figs 2–21 for NMR, high-resolution TOF-MS, and IR spectra of the products.

### Synthesis of **1**

A stirred mixture of **7** (68.8 mg, 0.151 mmol), Pd/C (ASCA-2, 49.9 mg, 0.021 mmol Pd), aniline (3.9 mg, 0.042 mmol) in DMAc (1.6 ml) was treated with hydrogen atmosphere (1 atm) at room temperature for 2 h (Supplementary Table 2 Entry 7). The reaction mixture was filtered through a membrane filter (GE Healthcare Puradisc 13, 0.45 μm) and the filter was washed with a small amount (*ca*. 0.2 ml) of DMAc to give a clear solution (total amount of the resulting solution was ca. 1.6 ml). To the solution was added 1,4-bis(trimethylsilyl)benzene (18.7 mg, 0.084 mmol) as an internal standard, a trace amount of Cr(acac)_3_ as a relaxation agent and THF-*d*_*8*_ (0.3 ml) to give an NMR sample, which was subjected to ^1^H and ^29^Si NMR analysis showing the complete disappearance of **7** and the formation of **1** and **2** in 90% and 8% yields, respectively. **1**: ^1^H NMR (DMAc:THF-*d*_8_, 5:1 v/v, *δ*) 5.69 (s, 4H, O*H*); ^29^Si NMR (DMAc:THF-*d*_8_, 5:1 v/v, *δ*) –71.9; HRMS (ESI) *m/z* calcd for H_4_O_4_SiCl 130.9573 [*M*+Cl]^–^, found 130.9568.

### Synthesis of **2**

A stirred mixture of **8** (108.1 mg, 0.151 mmol), Pd/C (ASCA-2, 74.6 mg, 0.032 mmol Pd), aniline (4.4 mg, 0.047 mmol) in DMAc (1.6 ml) was treated with hydrogen atmosphere (1 atm) at room temperature for 2 h. The reaction mixture was treated and analyzed by ^1^H and ^29^Si NMR as described for **1**, showing the complete disappearance of **8** and the formation of **2** in 94% yields. **2**: ^1^H NMR (DMAc:THF-*d*_8_, 5:1 v/v, *δ*) 5.80 (s, 6H, O*H*); ^29^Si NMR (DMAc:THF-*d*_8_, 5:1 v/v, *δ*) –80.5; HRMS (ESI) *m/z* calcd for H_6_O_7_Si_2_Cl 208.9346 [*M*+Cl]^–^, found 208.9347.

### Synthesis of **4**

A stirred mixture of **9** (146.8 mg, 0.151 mmol), Pd/C (ASCA-2, 99.6 mg, 0.042 mmol Pd), aniline (2.9 mg, 0.032 mmol) in DMAc (1.6 ml) was treated with hydrogen atmosphere (1 atm) at room temperature for 2 h. The reaction mixture was treated and analyzed by ^1^H and ^29^Si NMR as described for **1**, showing the complete disappearance of **9** and the formation of linear trisiloxane **3** and cyclotrisiloxane **4** in 3% and 94% yields, respectively. **4**: ^1^H NMR (DMAc:THF-*d*_8_, 5:1 v/v, *δ*) 6.59 (s, 6H, O*H*); ^29^Si NMR (DMAc:THF-*d*_8_, 5:1 v/v, *δ*) –83.0; HRMS (ESI) *m/z* calcd for H_6_O_9_Si_3_Cl 268.9014 [*M*+Cl]^–^, found 268.9006.

### Data availability

X-ray and neutron crystallographic data have been deposited at The Cambridge Crystallographic Data Centre (CCDC), under the deposition numbers CCDC 1406687–1406689 and 1482884–1482886. These data can be obtained free of charge from The CCDC at www.ccdc.cam.ac.uk/data_request/cif. The data that support the findings of this study are available from the corresponding author upon reasonable request.

## Electronic supplementary material

Supplementary Information

Peer Review File
