# Supplementary material for: Non-aqueous selective synthesis of orthosilicic acid and its oligomers
Source: Nat Commun. 2017 Jul 26;8:140. doi: 10.1038/s41467-017-00168-5 (PMC5529440; doi:10.1038/s41467-017-00168-5)
Supplement: Supplementary file 1 — Supplementary Information [file 41467_2017_168_MOESM1_ESM.pdf]

File Name: Supplementary Information
Description: Supplementary Figures, Supplementary Tables, Supplementary Methods, and Supplementary References.

File Name: Peer Review File
Description:

Supplementary Methods

General Information. ^{1}H (600 MHz), ^{13}C NMR (151 MHz) and ^{29}Si NMR (119 MHz) spectra were measured on a Bruker AVANCEIII spectrometer equipped with a CryoProbe using $CDCl_3$ or THF-d_8 as a solvent. Chemical shifts (δ) are reported in parts per million (ppm) relative to an internal standard (1,4-bis(trimethylsilyl)benzene, 0.25 ppm for ^{1}H and –4.3 ppm for ^{29}Si for **1**, **2**, **4**, and **6**). In the case of ^{1}H, ^{13}C, and ^{29}Si NMR spectra of **7-9**, **10b**, **11b**, and **13b** tetramethylsilane in THF-d_8 (0.00 ppm) was used as an external standard. ^{1}H-^{29}Si long-range gHMBC spectra were acquired with pulse field gradients in absolute value mode. The spectral windows for ^{1}H and ^{29}Si domains were 12 and 200 ppm, respectively. The multiple-bond delay was adjusted to a coupling constant of 7 Hz. The data were collected in a 2048 × 512 matrix with four transients per t_1 increment. The recycle period was 2.14 s. Sine-bell window functions were applied before Fourier transformation in a 2048 × 1024 matrix. Standard procedures of the spectrometer software (Topspin ver. 3.2) were used.

High-resolution time-of-flight mass spectra of the products (electrospray-ionization (ESI) except for **10b**, for which atmospheric pressure chemical ionization (APCI) was used) were analyzed on a Bruker micrOTOF II spectrometer operating in negative (for **1**, **2**, **4**, and **6**) and positive (for **7-9**, **10b**, **11b**, and **13b**) ion modes. Infrared spectra (IR) were measured on a JASCO FT/IR-6800 spectrometer using a JASCO ATR PRO550S-S attachment. UV spectra were measured on a SCINCO Photodiode Array UV-Vis Spectrophotometer S-3100.

The Pd/C catalyst (ASCA-2 type) was obtained from N. E. CHEMCAT Co. Ltd. 1,4-Bis(trimethylsilyl)benzene, N-methylacetamide (MMAc), Hydranal Coulomat AK and Hydranal Coulomat CG-K were obtained from Aldrich Chemical Co. Hexaammonium heptamolybdate tetrahydrate, silicon standard solution (1000 ppm) for the molybdenum yellow method, benzyl alcohol, diethyl ether (super dehydrated grade), *N,N*-dimethylacetamide (DMAc, super dehydrated grade), *N,N*-dimethylformamide (super dehydrated grade), toluene (super dehydrated grade), triethylamine, acetonitrile (super dehydrated grade), ethyl acetate (super dehydrated grade), dimethyl sulfoxide (super dehydrated grade), sodium sulfate, dichloromethane (super dehydrated grade), methanol (super dehydrated grade), heptane (super dehydrated grade), potassium carbonate (anhydrous) and hydrochloric acid were obtained from Wako Pure Chemicals Ind., Ltd. Acetamide (Ac), tetramethylurea (TMU), diphenylamine, $SiCl_4$, 4-(dimethylamino)pyridine (DMAP), tetrabutylammonium chloride, tetrabutylammonium bromide, $Si(OCH_3)_4$, $Si(OCH_2CH_3)_4$, and diacetoxydimethylsilane were obtained from Tokyo Chemical Industry Co., Ltd. Tetrahydrofuran (THF, dehydrated stabilizer free Super Plus grade), hexane

(dehydrated Super grade), aniline and $CDCl_3$ were obtained from Kanto Chemical Co., Inc. THF-d_8 was obtained from Cambridge Isotope Laboratories, Inc. H_2 (G1 grade, 99.99999%) was obtained from Taiyo Nippon Sanso Corporation. D_2 (UHP grade, 99.995%) was obtained from Linde. $Cl_3SiOSiCl_3$ and $Cl_3SiOSiCl_2OSiCl_3$ were obtained from Gelest, Inc. The water content of DMAc (super dehydrated grade) was determined by Karl Fischer titration to be 8 ppm.

The Pd/C catalyst was dried at 120 °C for 48 h under vacuum. THF and hexane used in the hydrogenolysis reaction were dried and degassed by the Glass Contour solvent purification system. THF-d_8 was dried by refluxing over Na-benzophenone ketyl and distilled.

Synthesis of tetrakis(benzyloxy)silane 7. To a solution of benzyl alcohol (64.3 g, 595 mmol), Et_3N (60.2 g, 595 mmol) and DMAP (177 mg, 1.45 mmol) in CH_2Cl_2 (500 mL) was added $SiCl_4$ (24.6 g, 145 mmol) dropwise[1]. After stirring at 0 ºC for 3 h and at room temperature for 3 d, the volatiles were removed under vacuum leaving a colorless residue, which was diluted with hexane (1 L), washed with water (20 L) to remove triethylamine hydrochloride and other water-soluble impurities, and dried over Na_2SO_4. The solvent was removed under reduced pressure, and the residue was purified by Kugelrohr distillation (230 ºC, 10 Pa) to afford **7** (57.7 g, 87% yield) as a colorless solid.

$Si(OCH_2C_6H_5)_4$: ^{1}H NMR ($CDCl_3$, δ) 4.81 (s, 8H, OCH_2), 7.22 – 7.32 (20H, C_6H_5); ^{13}C NMR ($CDCl_3$, δ) 65.6 ($CH_2C_6H_5$), 126.9, 127.4, 128.3, 139.9; ^{29}Si NMR ($CDCl_3$, δ) –81.8; ^{1}H NMR (THF-d_8, δ) 4.83 (s, 8H, OCH_2), 7.15 – 7.32 (20H, C_6H_5); ^{13}C NMR (THF-d_8, δ) 66.1 ($CH_2C_6H_5$), 127.4, 127.9, 128.9, 141.2; ^{29}Si NMR (THF-d_8, δ) –81.8; HRMS (ESI) *m/z* calcd. for $C_{28}H_{32}NO_4Si$ 474.2095 $[M+NH_4]^+$, found 474.2091; elemental analysis calcd. for $C_{28}H_{28}O_4Si$ C 73.65, H 6.18, found C 73.70, H 5.99.

Synthesis of hexakis(benzyloxy)disiloxane 8. To a solution of benzyl alcohol (23.8 g, 221 mmol), Et_3N (22.3 g, 221 mmol) and DMAP (42.8 mg, 0.35 mmol) in CH_2Cl_2 (300 mL) was added $Cl_3SiOSiCl_3$ (9.9 g, 35 mmol) dropwise[1]. After stirring at 0 ºC for 2 h and at room temperature for 1 d, the volatiles were removed under vacuum leaving a colorless residue, which was diluted with hexane (1 L), washed with water (12 L) to remove triethylamine hydrochloride and other water-soluble impurities, and dried over Na_2SO_4. The solvent was removed under reduced pressure, and the residue was purified by a silica gel column chromatography (eluent: hexane/ethyl acetate, 100/0 - 80/20 (v/v)) to afford **8** (14.0 g, 56% yield) as a colorless liquid.

$(C_6H_5CH_2O)_3SiOSi(OCH_2C_6H_5)_3$: ^{1}H NMR ($CDCl_3$, δ) 4.84 (s, 12H, O$CH_2$), 7.22 – 7.38 (30H, C_6H_5); ^{13}C NMR ($CDCl_3$, δ) 65.5 ($CH_2C_6H_5$), 126.8, 127.3, 128.3, 139.8; ^{29}Si NMR ($CDCl_3$, δ) –

88.5; HRMS (ESI) *m/z* calcd. for $C_{42}H_{46}NO_7Si_2$ 732.2807 $[M+NH_4]^+$, found 732.2808; elemental analysis calcd. for $C_{42}H_{42}O_7Si_2$ C 70.56, H 5.92, found C 70.59, H 5.93.

Synthesis of octakis(benzyloxy)trisiloxane 9. To a solution of benzyl alcohol (21.9 g, 203 mmol), Et_3N (20.6 g, 203 mmol) and DMAP (30.8 mg, 0.25 mmol) in CH_2Cl_2 (250 mL) was added $Cl_3SiOSiCl_2OSiCl_3$ (10.0 g, 25 mmol) dropwise[1]. After stirring at 0 ºC for 3 h and at room temperature for 1 d, the volatiles were removed under vacuum leaving a colorless residue, which was diluted with hexane (1 L), washed with water (12 L) to remove triethylamine hydrochloride and other water-soluble impurities, and dried over Na_2SO_4. The solvent was removed under reduced pressure, and the residue was purified by a silica gel column chromatography (eluent: hexane/ethyl acetate, 100/0 - 80/20 (v/v)) to afford **9** (13.8 g, 57% yield) as a colorless liquid.
$(C_6H_5CH_2O)_3SiOSi(OCH_2C_6H_5)_2OSi(OCH_2C_6H_5)_3$: ^1H NMR (THF-d_8, δ) 4.87 (12H, OCH_2), 4.88 (4H, OCH_2), 7.12 – 7.40 (40H, C_6H_5); ^{13}C NMR (THF-d_8, δ) 66.16 ($CH_2C_6H_5$), 66.19 ($CH_2C_6H_5$), 127.40, 127.46, 127.9, 129.0, 140.9, 141.0; ^{29}Si NMR (THF-d_8, δ) –95.4 (Si, *Si*$(OCH_2C_6H_5)_2$) –88.5 (2 Si, *Si*$(OCH_2C_6H_5)_3$); HRMS (ESI) *m/z* calcd. for $C_{56}H_{60}NO_{10}Si_3$ 990.3520 $[M+NH_4]^+$, found 990.3513; elemental analysis calcd. for $C_{56}H_{56}O_{10}Si_3$ C 69.11, H 5.80, found C 69.08, H 5.59.

A representative procedure for the hydrogenolysis reaction of tetrakis(benzyloxy)silane 7 (Supplementary Table 2, Entry 7). A stirred mixture of **7** (68.8 mg, 0.151 mmol), Pd/C (ASCA-2, 49.9 mg, 0.021 mmol Pd), aniline (3.9 mg, 0.042 mmol) in DMAc (1.6 mL) was treated with hydrogen atmosphere (1 atm) at room temperature for 2 h. The reaction mixture was filtered through a membrane filter (GE Healthcare Puradisc 13, 0.45 μm) and the filter was washed with a small amount (ca. 0.2 mL) of DMAc to give a clear solution (total amount of the resulting solution was ca. 1.6 mL). To the solution was added 1,4-bis(trimethylsilyl)benzene (18.7 mg, 0.084 mmol) as an internal standard, a trace amount of $Cr(acac)_3$ as a relaxation agent and THF-d_8 (0.3 mL) to give an NMR sample, which was subjected to ^1H and ^{29}Si NMR analysis showing the complete disappearance of the starting compound and the formation of **1** and **2** in 90% and 8% yields, respectively.
$Si(OH)_4$: ^1H NMR (DMAc:THF-d_8, 5:1 v/v, δ) 5.69 (s, 4H, OH); ^{29}Si NMR (DMAc:THF-d_8, 5:1 v/v, δ) –71.9; HRMS (ESI) *m/z* calcd for H_4O_4SiCl 130.9573 $[M+Cl]^-$, found 130.9568. $(HO)_3SiOSi(OH)_3$: ^1H NMR (DMAc:THF-d_8, 5:1 v/v, δ) 5.80 (s, 6H, OH); ^{29}Si NMR (DMAc:THF-d_8, 5:1 v/v, δ) –80.5.

Supplementary Table 1. Solvent effects in the hydrogenolysis reaction of 7.[a]

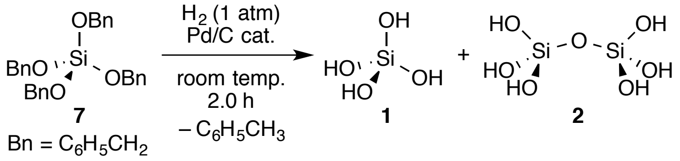

Entry	Solvent	Conversion[b] (%)	Yield (%)[c]	
			**1**	**2**
1	THF	100	0	0
2	Hexane	100	0	0
3	Toluene	100	0	0
4	$CH_3CO_2C_2H_5$	100	1	0
5	CH_2Cl_2	100	0	0
6	CH_3CN	100	0	0
7	DMSO	100	0	0
8	DMF	100	0	5
9	TMU	100	0	25
10	DMAc	100	13	34
11	Ac/DMAc (25/75)	100	42	38
12	MMAc/DMAc (90/10)	100	85	13

[a]Reaction conditions: **7** (0.15 mmol) and Pd/C (ASCA-2 type, 0.012 mmol Pd) were stirred under hydrogen atmosphere (1 atm) at room temperature for 2.0 h in various solvents (1.6 mL). [b]Conversion of **7**. [c]Yields based on **7** were determined by the integral values of ^{29}Si NMR signals of the reaction mixture using 1,4-bis(trimethylsilyl)benzene as an internal standard.

Supplementary Table 2. Optimization of the reaction conditions for the hydrogenolysis reaction of 7.[a]

Entry	Pd (mmol)	Aniline/Pd[b]	Solvent	Conversion[c] (%)	Yield (%)[d]	
					**1**	**2**
1[e]	0.012	0	DMAc	100	13	34
2	0.012	1.0	DMAc	100	57	26
3	0.012	1.5	DMAc	100	75	8
4	0.012	2.0	DMAc	100	45	3
5	0.015	2.0	DMAc	100	66	4
6	0.018	2.0	DMAc	100	77	5
7	0.021	2.0	DMAc	100	90	8
8[f]	0.012	0	MMAc/DMAc (90/10)	100	85	13
9	0.012	1.0	MMAc/DMAc (90/10)	100	78	0
10[g]	0.018	1.0	MMAc/DMAc (90/10)	100	96	2

[a]Reaction conditions: **7** (0.15 mmol) and Pd/C (ASCA-2 type) were stirred under hydrogen atmosphere (1 atm) at room temperature for 2.0 h in DMAc or MMAc-DMAc mixed solvent (1.6 mL) in the presence of aniline. [b]Molar ratio of aniline/Pd. [c]Conversion of **7**. [d]Yields based on **7** were determined by the integral values of ^{29}Si NMR signals of the reaction mixture using 1,4-bis(trimethylsilyl)benzene as an internal standard. [e]The data from Supplementary Table 1, Entry 10. [f]The data from Supplementary Table 1, Entry 12. [g]The yields of **1** and **2** are the average of three experiments.

A representative procedure for the hydrogenolysis reaction of hexakis(benzyloxy)disiloxane 8.

A stirred mixture of **8** (108.1 mg, 0.151 mmol), Pd/C (ASCA-2, 74.6 mg, 0.032 mmol Pd), aniline (4.4 mg, 0.047 mmol) in DMAc (1.6 mL) was treated with hydrogen atmosphere (1 atm) at room temperature for 2 h. The reaction mixture was filtered through a membrane filter (GE Healthcare Puradisc 13, 0.45 μm) and the filter was washed with a small amount (ca. 0.2 mL) of DMAc to give a clear solution (total amount of the resulting solution was ca. 1.6 mL). To the solution was added 1,4-bis(trimethylsilyl)benzene (17.0 mg, 0.076 mmol) as an internal standard, a trace amount of $Cr(acac)_3$ as a relaxation agent and THF-d_8 (0.3 mL) to give an NMR sample, which was subjected to ^{1}H and ^{29}Si NMR analysis showing the complete disappearance of the starting compound and the formation of **2** in 94% yields.

$(HO)_3SiOSi(OH)_3$: ^{1}H NMR (DMAc:THF-d_8, 5:1 v/v, δ) 5.80 (s, 6H, O*H*); ^{29}Si NMR (DMAc:THF-d_8, 5:1 v/v, δ) –80.5; HRMS (ESI) *m/z* calcd for $H_6O_7Si_2Cl$ 208.9346 [*M*+Cl]$^-$, found 208.9347.

A representative procedure for the hydrogenolysis reaction of octakis(benzyloxy)trisiloxane 9.

A stirred mixture of **9** (146.8 mg, 0.151 mmol), Pd/C (ASCA-2, 99.6 mg, 0.042 mmol Pd), aniline (2.9 mg, 0.032 mmol) in DMAc (1.6 mL) was treated with hydrogen atmosphere (1 atm) at room temperature for 2 h. The reaction mixture was filtered through a membrane filter (GE Healthcare Puradisc 13, 0.45 μm) and the filter was washed with a small amount (ca. 0.2 mL) of DMAc to give a clear solution (total amount of the resulting solution was ca. 1.6 mL). To the solution was added 1,4-bis(trimethylsilyl)benzene (17.2 mg, 0.077 mmol) as an internal standard, a trace amount of $Cr(acac)_3$ as a relaxation agent and THF-d_8 (0.3 mL) to give an NMR sample, which was subjected to ^{1}H and ^{29}Si NMR analysis showing the complete disappearance of the starting compound and the formation of linear trisiloxane **3** and cyclotrisiloxane **4** in 3% and 94% yields. The reaction using a larger amount of aniline (3.9 mg) under otherwise the same reaction conditions afforded **3** and **4** in respectively 15% and 79% yields, and the following NMR data were taken using this sample.

$(HO)_3SiOSi(OH)_2OSi(OH)_3$: ^{1}H NMR (DMAc:THF-$d_8$, 5:1 v/v, δ) 6.02 (s, 2H, O*H*) 6.13 (s, 6H, O*H*); ^{29}Si NMR (DMAc:THF-d_8, 5:1 v/v, δ) –89.9 (Si, *Si*$(OH)_2$) –80.8 (2 Si, *Si*$(OH)_3$). $[Si(OH)_2O]_3$: ^{1}H NMR (DMAc:THF-d_8, 5:1 v/v, δ) 6.59 (s, 6H, O*H*); ^{29}Si NMR (DMAc:THF-d_8, 5:1 v/v, δ) –83.0; HRMS (ESI) *m/z* calcd for $H_6O_9Si_3Cl$ 268.9014 [*M*+Cl]$^-$, found 268.9006.

A representative procedure for the hydrolysis of $Si(OCH_3)_4$ forming $Si(OH)_4$.

To a stirred mixture of $Si(OCH_3)_4$ (20.7 mg, 136 μmol), H_2O (270 μL, 15.0 mmol) and DMAc (723 μL) in a 10 mL Teflon-coated glass vial were added a DMAc solution of HCl (1.0 M, 1.40 μL, 1.40

μmol). The mixture was stirred at room temperature for 30 min, and then the acid was neutralized by adding a DMAc solution of Et_3N (0.27 M, 5.00 μL, 1.35 μmol). A 250 μL solution was taken from the resulting mixture to prepare an NMR sample, to which was added a DMAc solution of 1,4-bis(trimethylsilyl)- benzene (0.100 M, 100 μL, 10.0 μmol) as an internal standard, a trace amount of $Cr(acac)_3$ as a relaxation agent and THF-d_8 (100 μL). The NMR sample was analyzed by ^{29}Si NMR spectroscopy showing the formation of **1** in quantitative yield (Supplementary Fig. 7).

Synthesis of tetrakis((methoxydimethylsilyl)oxy)silane 10b.

A stirred mixture of **7** (71 mg, 0.155 mmol), Pd/C (ASCA-2, 50 mg, 0.021 mmol Pd), aniline (3.9 mg, 0.042 mmol) in DMAc (1.6 mL) was treated with hydrogen atmosphere (1 atm) at room temperature for 2 h. The reaction mixture was filtered through a membrane filter (GE Healthcare Puradisc 13, 0.45 μm) and the filter was washed with a small amount (ca. 0.3 mL) of DMAc to give a clear solution. DMAc (0.087 mL) solution of diacetoxydimethylsilane (213 mg, 1.21 mmol) and aniline (227 mg, 2.43 mmol) was added to the filtrate dropwise at –40 °C under Ar atmosphere, followed by stirring at room temperature for 15 min. To a resulting mixture was added MeOH (78 mg, 2.42 mmol) and the mixture was shook several times by hand. After standing for 10 min, the product was extracted with heptane (2.25 mL × 3) from the DMAc solution. Anhydrous K_2CO_3 (1.00 g, 7.24 mmol) was added to the separated heptane solution to remove dissolved trace amount of acetic acid, and the mixture was stirred for 30 min and then filtered through a membrane filter (GE Healthcare Puradisc 13, 0.45 μm). After the removal of volatiles under vacuum, 1,4-bis(trimethylsilyl)benzene (4.7 mg, 0.021 mmol) as an internal standard, a trace amount of $Cr(acac)_3$ as a relaxation agent and THF-d_8 (0.45 mL) were added to the residue to give an NMR sample, which was subjected to ^{1}H and ^{13}C and ^{29}Si NMR analysis showing the complete disappearance of the starting compound and the formation of **10b** and **13b** in 64% and 11% yields, respectively.

$Si\{OSi(CH_3)_2(OCH_3)\}_4$: ^{1}H NMR (THF-$d_8$, δ) 0.12 (24H, Si(C$H_3$)$_2$), 3.48 (12H, OC$H_3$); ^{13}C NMR (THF-$d_8$, δ) –1.7 (Si($CH_3$)$_2$), 50.0 (O$CH_3$); ^{29}Si NMR (THF-$d_8$, δ) –108.1 (1Si, Si\{OSi(CH$_3$)$_2$(OCH$_3$)\}$_4$), –10.8 (4Si, Si(CH$_3$)$_2$(OCH$_3$)); HRMS (APCI) m/z calcd. for $C_{12}H_{40}NO_8Si_5$ $[M+NH_4]^+$ 466.1595, found 466.1583.

Synthesis of 2,2,4,4,6,6-hexakis((methoxydimethylsilyl)oxy)cyclotrisiloxane 11b.

A stirred mixture of **9** (147 mg, 0.151 mmol), Pd/C (ASCA-2, 99.6 mg, 0.042 mmol Pd), aniline (2.9 mg, 0.032 mmol) in DMAc (1.6 mL) was treated with hydrogen atmosphere (1 atm) at room temperature for 2 h. The reaction mixture was filtered through a membrane filter (GE Healthcare

Puradisc 13, 0.45 μm) and the filter was washed with a small amount (ca. 0.3 mL) of DMAc to give a clear solution. DMAc (0.093 mL) solution of diacetoxydimethylsilane (317 mg, 1.80 mmol) and aniline (339 mg, 3.64 mmol) was added to the filtrate dropwise at –40 ˚C under Ar atmosphere, followed by stirring at room temperature for 15 min. To a resulting mixture was added MeOH (122 mg, 3.82 mmol) and the mixture was shook several times by hand. After standing for 10 min, the product was extracted with heptane (2.25 mL × 3) from the DMAc solution. Anhydrous K_2CO_3 (1.00 g, 7.235 mmol) was added to the separated heptane solution to remove dissolved trace amount of acetic acid, and the mixture was stirred for 30 min and then filtered through a membrane filter (GE Healthcare Puradisc 13, 0.45 μm). After the removal of volatiles under vacuum, 1,4-bis(trimethylsilyl)benzene (5.3 mg, 0.024 mmol) as an internal standard, a trace amount of $Cr(acac)_3$ as a relaxation agent and THF-d_8 (0.46 mL) were added to the residue to give an NMR sample, which was subjected to ^{1}H and ^{13}C and ^{29}Si NMR analysis showing the complete disappearance of the starting compound and the formation of **11b** in 78% yield.

$[Si\{OSi(CH_3)_2(OCH_3)\}_2O]_3$: ^{1}H NMR (THF-$d_8$, δ) 0.14 (36H, Si(C$H_3$)$_2$), 3.49 (18H, OC$H_3$); ^{13}C NMR (THF-$d_8$, δ) –1.7 (Si($CH_3$)$_2$), 50.1 (O$CH_3$); ^{29}Si NMR (THF-$d_8$, δ) –102.1 (3Si, Si\{OSi(CH$_3$)$_2$(OCH$_3$)\}$_2$), –9.5 (6Si, Si(CH$_3$)$_2$(OCH$_3$)); HRMS (ESI) m/z calcd. for $C_{18}H_{54}NaO_{15}Si_9$ 785.1278 $[M+Na]^+$, found 785.1278.

Synthesis of 2,2,4,4-tetrakis((methoxydimethylsilyl)oxy)-6,6-dimethylcyclotrisiloxane 13b.

A stirred mixture of **8** (108 mg, 0.151 mmol), Pd/C (ASCA-2, 74.6 mg, 0.032 mmol Pd), aniline (4.4 mg, 0.047 mmol) in DMAc (1.6 mL) was treated with hydrogen atmosphere (1 atm) at room temperature for 2 h. The reaction mixture was filtered through a membrane filter (GE Healthcare Puradisc 13, 0.45 μm) and the filter was washed with a small amount (ca. 0.3 mL) of DMAc to give a clear solution. DMAc (0.093 mL) solution of diacetoxydimethylsilane (317.7 mg, 1.803 mmol) and aniline (336 mg, 3.61 mmol) was added to the filtrate dropwise at –40 ˚C under Ar atmosphere, followed by stirring at room temperature for 15 min. To a resulting mixture was added MeOH (117 mg, 3.66 mmol) and the mixture was shook several times by hand. After standing for 10 min, the product was extracted with heptane (2.25 mL × 3) from the DMAc solution. Anhydrous K_2CO_3 (1.00 g, 7.24 mmol) was added to the separated heptane solution to remove dissolved trace amount of acetic acid, and the mixture was stirred for 30 min and then filtered through a membrane filter (GE Healthcare Puradisc 13, 0.45 μm). After the removal of volatiles under vacuum, 1,4-bis(trimethylsilyl)benzene (5.0 mg, 0.022 mmol) as an internal standard, a trace amount of $Cr(acac)_3$ as a relaxation agent and THF-d_8 (0.45 mL) were added to the residue to give an NMR sample, which was subjected to ^{1}H and ^{13}C and ^{29}Si NMR analysis showing the complete

disappearance of the starting compound and the formation of **13b** in 92% yield.

13b: ^{1}H NMR (THF-d_8, δ) 0.12 (24H, Si(C*H*$_3$)$_2$(OCH$_3$)), 0.21 (6H, OSi(C*H*$_3$)$_2$O), 3.48 (12H, OC*H*$_3$); ^{13}C NMR (THF-d_8, δ) –1.8 (Si(*C*H$_3$)$_2$(OCH$_3$)), 0.4 (OSi(*C*H$_3$)$_2$O), 50.0 (O*C*H$_3$); ^{29}Si NMR (THF-d_8, δ) –101.6 (2Si, *Si*{OSi(CH$_3$)$_2$(OCH$_3$)}$_2$), –9.9 (4Si, *Si*(CH$_3$)$_2$(OCH$_3$)), –6.4 (1Si, O*Si*(CH$_3$)$_2$O); HRMS (ESI) m/z calcd. for $C_{14}H_{42}NaO_{11}Si_7$ 605.1004 [M+Na]$^+$, found 605.1001.

Single Crystal Preparations.

Preparation of single crystals of hydrogen-bonded complexes [Si(OH)$_4$·2(^{n}Bu$_4$NCl)], [Si(OD)$_4$·2(^{n}Bu$_4$NCl)] and [Si(OH)$_4$·2(^{n}Bu$_4$NBr)].

A solution of Si(OH)$_4$ was prepared as described above from **1** (69.1 mg, 0.151 mmol), Pd/C (ASCA-2, 49.7 mg, 0.021 mmol Pd), aniline (2.5 mg, 0.027 mmol) in tetramethylurea (TMU, 1.6 mL) instead of DMAc. To the resulting clear solution was added ^{n}Bu$_4$NCl (83.7 mg, 0.301 mmol) in TMU (1.6 mL) and diethyl ether (1.5 g). The mixture in a small glass vial was put in a larger glass vial containing diethyl ether (2.5 g) and the larger glass vial was closed with a cap. After a few days, single crystals of [Si(OH)$_4$·2(^{n}Bu$_4$NCl)] were formed, which were separated by decantation, washed with diethyl ether, and dried under vacuum (76 mg, 77% yield). Single crystals of [Si(OD)$_4$·2(^{n}Bu$_4$NCl)] were similarly obtained by performing the reaction under D_2 instead of H_2. Also single crystals of [Si(OH)$_4$·2(^{n}Bu$_4$NBr)] were obtained by performing the crystallization in the presence of ^{n}Bu$_4$NBr instead of ^{n}Bu$_4$NCl.

Preparation of single crystals of hydrogen-bonded complex [(HO)$_3$SiOSi(OH)$_3$·2(^{n}Bu$_4$NCl)].

A solution of (HO)$_3$SiOSi(OH)$_3$ was prepared as described above from **8** (107.4 mg, 0.150 mmol), Pd/C (ASCA-2, 74.5 mg, 0.032 mmol Pd), aniline (2.9 mg, 0.032 mmol) in tetramethylurea (TMU, 1.6 mL) instead of DMAc. To the resulting clear solution was added ^{n}Bu$_4$NCl (84.7 mg, 0.305 mmol) in TMU (1.6 mL) and diethyl ether (3.7 g). Keeping the solution at –40 °C for a few days afforded single crystals of [(HO)$_3$SiOSi(OH)$_3$·2(^{n}Bu$_4$NCl)], which were separated by decantation, washed with diethyl ether, and dried under vacuum (90 mg, 82% yield).

Preparation of single crystals of {[Si(OH)$_2$O]$_3$·2TMU}.

A solution of **4** was prepared as described above from **9** (146.9 mg, 0.151 mmol), Pd/C (ASCA-2, 99.4 mg, 0.042 mmol Pd), aniline (2.0 mg, 0.021 mmol) in tetramethylurea (TMU, 1.6 mL) instead of DMAc. The resulting clear solution was diluted with TMU (14.4 mL). The mixture was frozen with liquid nitrogen and then subjected to freeze-drying at –40 °C, 2 Pa for one week. During this

period, the residue was partially liquefied and crystallization took place to give colorless crystalline [**4**·2TMU] (74 mg, quantitative).

Preparation of single crystals of hydrogen-bonded complexes {[Si(OH)$_2$O]$_4$·4(^{n}Bu$_4$NCl)·2TMU}.

Hydrogenolysis reaction of **8** (107.7 mg, 0.151 mmol) was performed by using Pd/C (ASCA-2, 43.0 mg, 0.018 mmol Pd) in tetramethylurea (TMU, 1.6 mL) instead of DMAc, giving a mixture mainly containing **2** (40%), **4** (15%), and **6** (17%). To the resulting clear solution was added ^{n}Bu$_4$NCl (83.5 mg, 0.300 mmol) in TMU (1.6 mL) and diethyl ether (2.3 g). Keeping the solution at room temperature for a few days afforded single crystals of [**6**·4(^{n}Bu$_4$NCl)·2TMU], which were separated by decantation, washed with diethyl ether, and dried under vacuum (52 mg, 42% yield).

The following NMR data (Supplementary Fig. 6) were obtained by dissolving [**6**·4(^{n}Bu$_4$NCl)·2TMU] in a mixed solvent (DMF-d_7:THF-d_8 = 8:1 v/v). ^{1}H NMR (DMF-d_7:THF-d_8, 8:1 v/v, δ) 6.44 (s, 8 H, O*H*); ^{29}Si NMR (DMF-d_7:THF-d_8, 8:1 v/v, δ) –89.1 (Si, *Si*(OH)$_2$); HRMS (ESI) *m/z* calcd for $H_8O_{12}Si_4Cl$ 346.8787 [*M*+Cl]$^-$, found 346.8793.

Single crystal X-ray diffraction analysis of [Si(OH)$_4$·2(^{n}Bu$_4$NCl)], [Si(OH)$_4$·2(^{n}Bu$_4$NBr)], [(HO)$_3$SiOSi(OH)$_3$·2(^{n}Bu$_4$NCl)], {[Si(OH)$_2$O]$_3$·2TMU} and {[Si(OH)$_2$O]$_4$·4(^{n}Bu$_4$NCl)·2TMU}.

Data collection was performed on a Bruker Apex II diffractometer (Mo Kα radiation, graphite monochromator) except for [**4**·2TMU], for which a Rigaku XtaLAB P100 diffractometer (Mo Kα radiation, graphite monochromator) was used. Data were corrected for absorption. The structures were solved by the Patterson method[2] or direct method[3] for [**4**·2TMU]. Structure refinement was carried out by full-matrix least squares on F^2. Structure solution and refinement were performed using CrystalStructure software package with SHELX2013 program[4]. CCDC 1406687, 1406688, 1482884 - 1482886 contain the supplementary crystallographic data for [**1**·2(^{n}Bu$_4$NCl)], [**1**·2(^{n}Bu$_4$NBr)], [**2**·2(^{n}Bu$_4$NCl)], [**4**·2TMU] and [**6**·4(^{n}Bu$_4$NCl)·2TMU], respectively. These data can be obtained free of charge from The Cambridge Crystallographic Data Centre at www.ccdc.cam.ac.uk/data_request/cif.

Single crystal neutron diffraction analysis of [Si(OH)$_4$·2(^{n}Bu$_4$NCl)].

Single-crystal neutron diffraction data were collected using the single-crystal diffractometer SENJU[5] at the beamline BL18 of the Materials and Life-Science Facility (MLF), Japan Proton Accelerator Research Complex (J-PARC), using the wavelength-resolved time-of-flight Laue diffraction method for wavelength ranging from 0.4 to 4.4 Å (1st frame) and 4.6 to 8.8 Å (2nd frame).

Because the crystal of $[Si(OH)_4 \cdot 2(^nBu_4NCl)]$ is unstable in air, a single crystal of $[Si(OH)_4 \cdot 2(^nBu_4NCl)]$ ($2.0 \times 1.5 \times 0.5$ mm^3) was coated with the Apiezon N grease and then mounted on an aluminum pin and attached to a closed-cycle helium cryostat with a two-axes goniometer. Diffraction data was collected at 140 K under vacuum conditions using 37 two-dimensional scintillation detectors. Diffraction measurements with 12 crystal orientations at the 1st frame wavelength and 6 orientations at the 2nd frame wavelength were conducted. Measurement time for one crystal orientation was 4 h for the 1st frame and 1 h for the 2nd frame. The accelerator power was 300 kW. Data reduction was performed using the program STARGazer[6] to obtain hkl indexes and the corresponding integrated intensities of reflections corrected for the detector efficiency and Lorentz factor. The minimum value of the d-spacing of processed Bragg reflections was 0.7 Å. Numbers of total reflections and unique reflections were 16780 and 5239 respectively. A least-squares refinement of the atomic coordination parameters, scaling factors for each crystal orientations and parameters for the extinction were performed using JANA2006[7] with $I > 3\sigma(I)$ reflections. Atomic coordinates resulting from X-ray crystal structure analysis at 103 K were used as an initial structural model. At first, the structure refinement was carried out with a structural model in which hydrogen atoms bonding to the oxygen atoms of the $Si(OH)_4$ moiety of $[Si(OH)_4 \cdot 2(^nBu_4NCl)]$ were excluded and then a difference-Fourier (Fobs-Fcalc) map by program Olex-2[8] with the contour level –7.0. As a result, four strong negative peaks derived from the hydrogen atoms of the $Si(OH)_4$ moiety were clearly observed (Fig. 2c). Finally, structural parameters, positional parameters and anisotropic atomic displacement parameters of all hydrogen atoms including the hydrogen atoms of the $Si(OH)_4$ and non-hydrogen atoms were refined without structural constrain or restrain. The final R-factor was 0.1081. CCDC 1406689 contains the supplementary crystallographic data for this study.

Pulse delay time determination for the quantitative analysis by ^{29}Si NMR spectroscopy.

To ensure quantitative analysis by ^{29}Si NMR spectroscopy, at the beginning of the study, we determined the pulse delay time sufficient to quantify the product yields. ^{29}Si NMR spectra of a mixture of tetrakis(benzyloxy)silane **7** and the internal standard 1,4-bis(trimethylsilyl)benzene (molar ratio 2.00 : 1.00) in THF-d_8 with a trace amount of $Cr(acac)_3$ were measured with various pulse delay times 2.0, 5.0, 10.0, 20.0 and 40.0 sec (Supplementary Fig. 1 and Supplementary Table 3). From this experiment, 10.0 sec was adopted as the pulse delay time for quantitative analysis.

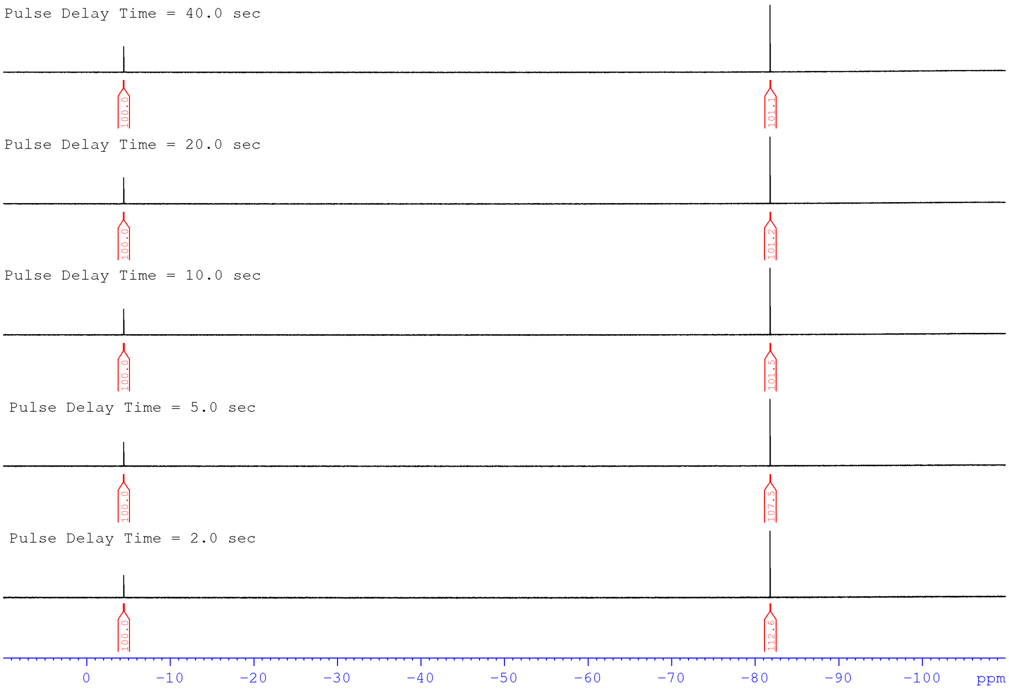

Supplementary Figure 1 | ^{29}Si NMR spectra of a mixture of tetrakis(benzyloxy)silane and 1,4-bis(trimethylsilyl)benzene with different pulse delay times.

Supplementary Table 3. Integral values of ^{29}Si NMR signals of 1,4-bis(trimethyl-silyl)benzene and tetrakis(benzyloxy)silane with different pulse delay times.

Entry	Pulse delay time (sec)	relative signal intensity	
		1,4-bis(trimethylsilyl)benzene	tetrakis(benzyloxy)silane
1	2.0	100.0	112.6
2	5.0	100.0	107.5
3	10.0	100.0	101.5
4	20.0	100.0	101.2
5	40.0	100.0	101.1

Comparison of the total yields of 1, 2, 3 and 4 determined by the ^{29}Si NMR spectroscopy and the molybdenum yellow method.

The DMAc solutions prepared by the hydrogenolysis reaction of **7-9** as described above were analyzed by the molybdenum yellow method (under the conditions of Japanese Industrial Standards, JIS). The results are summarized in Supplementary Table 4 together with the yields determined by ^{29}Si NMR spectroscopy.

Supplementary Table 4. Total yields of 1, 2, 3 and 4 determined by the ^{29}Si NMR spectroscopy and the molybdenum yellow method

Starting material	NMR yield (%)					Mo yellow method	
	**1**	**2**	**3**	**4**	**1**+**2**+**3**+**4**	yield (%)	concentrations (ppm)
**7**	90	8	1	0	99	95	8007
**8**	0	94	3	1	98	90	15376
**9**	0	0	3	94	97	93	24611

Spectra of products

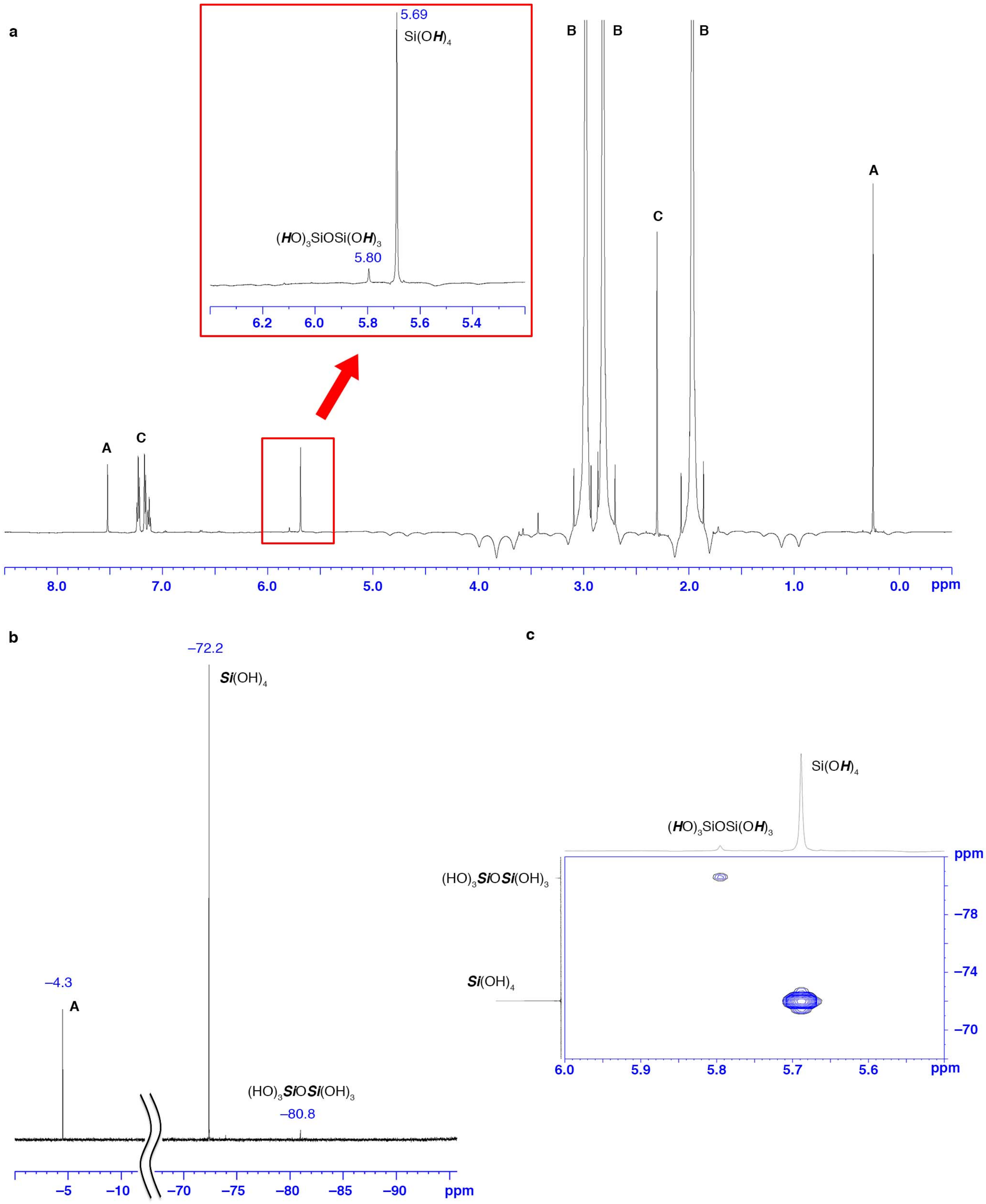

Supplementary Figure 2 | NMR spectra of the reaction mixture of the hydrogenolysis of 7. (**a**) ^{1}H NMR spectrum of the reaction mixture (Supplementary Table 2, entry 7). Signals of **1** and **2** are clearly observed. Other signals: **A** belong to the standard compound 1,4-{$(CH_3)_3Si$}$_2C_6H_4$; **B** belong to the solvent (DMAc, $CH_3CON(CH_3)_2$); **C** belong to toluene, which is the co-product of the hydrogenolysis reaction. (**b**) ^{29}Si NMR spectrum of the reaction mixture (Supplementary Table 2, entry 10). A signal of **1** is clearly observed together with a weak signal of **2**. **A** is the signal of the standard compound 1,4-{$(CH_3)_3Si$}$_2C_6H_4$. (**c**) ^{1}H-^{29}Si heteronuclear multiple-bond correlation (HMBC) NMR spectrum of the reaction mixture (Supplementary Table 2, entry 7). Cross peaks are clearly observed between respective ^{1}H and ^{29}Si signals of **1** and **2**. Details of the sample preparation are shown in the Supplementary Information.

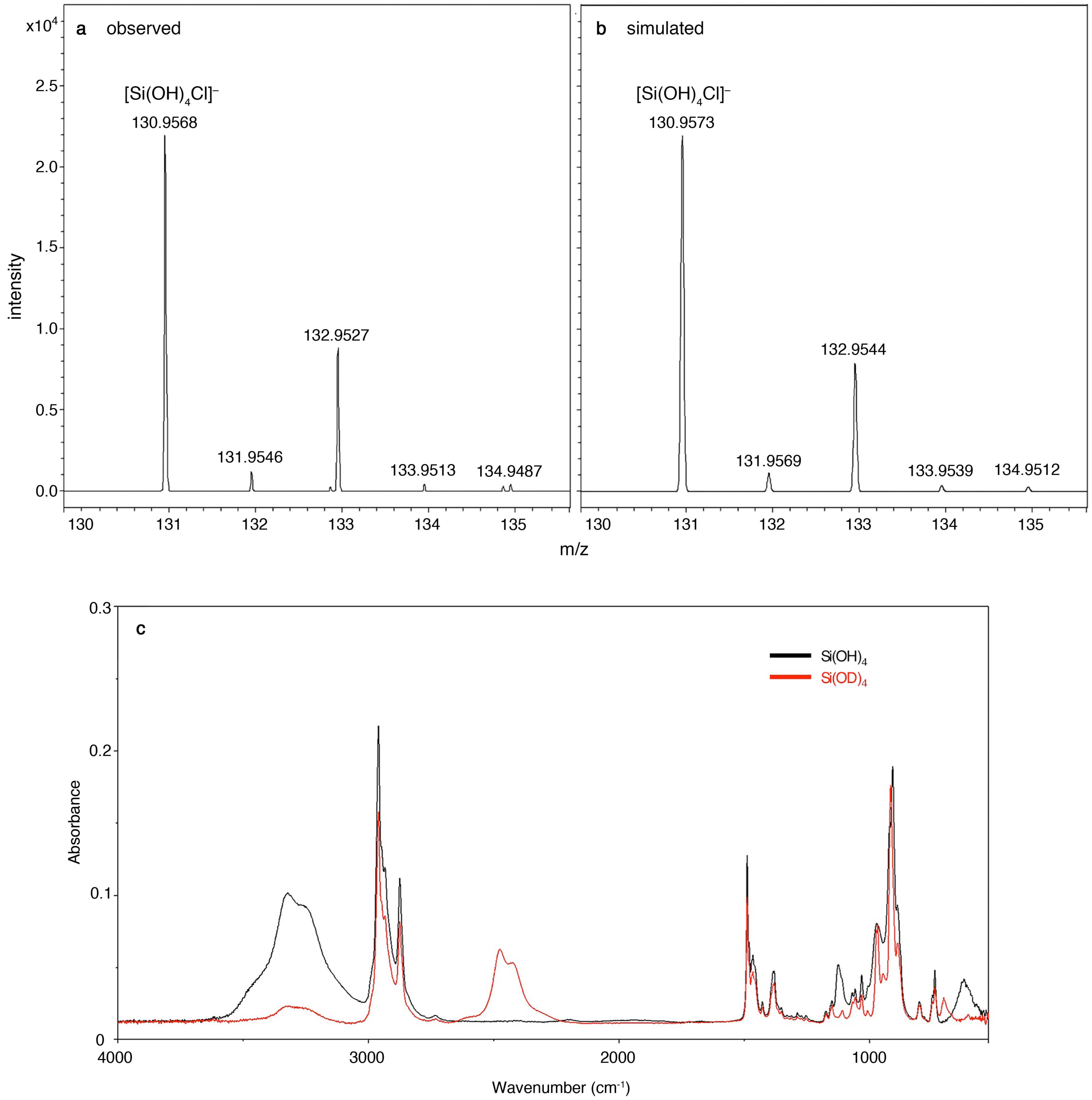

Supplementary Figure 3 | High-resolution TOF-MS and IR spectra of orthosilicic acid. (a) High-resolution TOF-MS spectrum of **1** obtained in the negative ion mode clearly showing $[Si(OH)_4{+}Cl]^-$ signal. (**b**) A simulated isotope pattern for $[Si(OH)_4{+}Cl]^-$. The observed spectrum is in good agreement with the simulated spectrum. (**c**) IR spectra of crystals of $[Si(OH)_4 \cdot 2(^n Bu_4NCl)]$ (black line) and $[Si(OD)_4 \cdot 2(^n Bu_4NCl)]$ (red line). The OH stretching band (broad absorption around 3300 cm^{-1}) for $Si(OH)_4$ and the OD stretching band (broad absorption around 2400 cm^{-1}) for $Si(OD)_4$ are clearly observed.

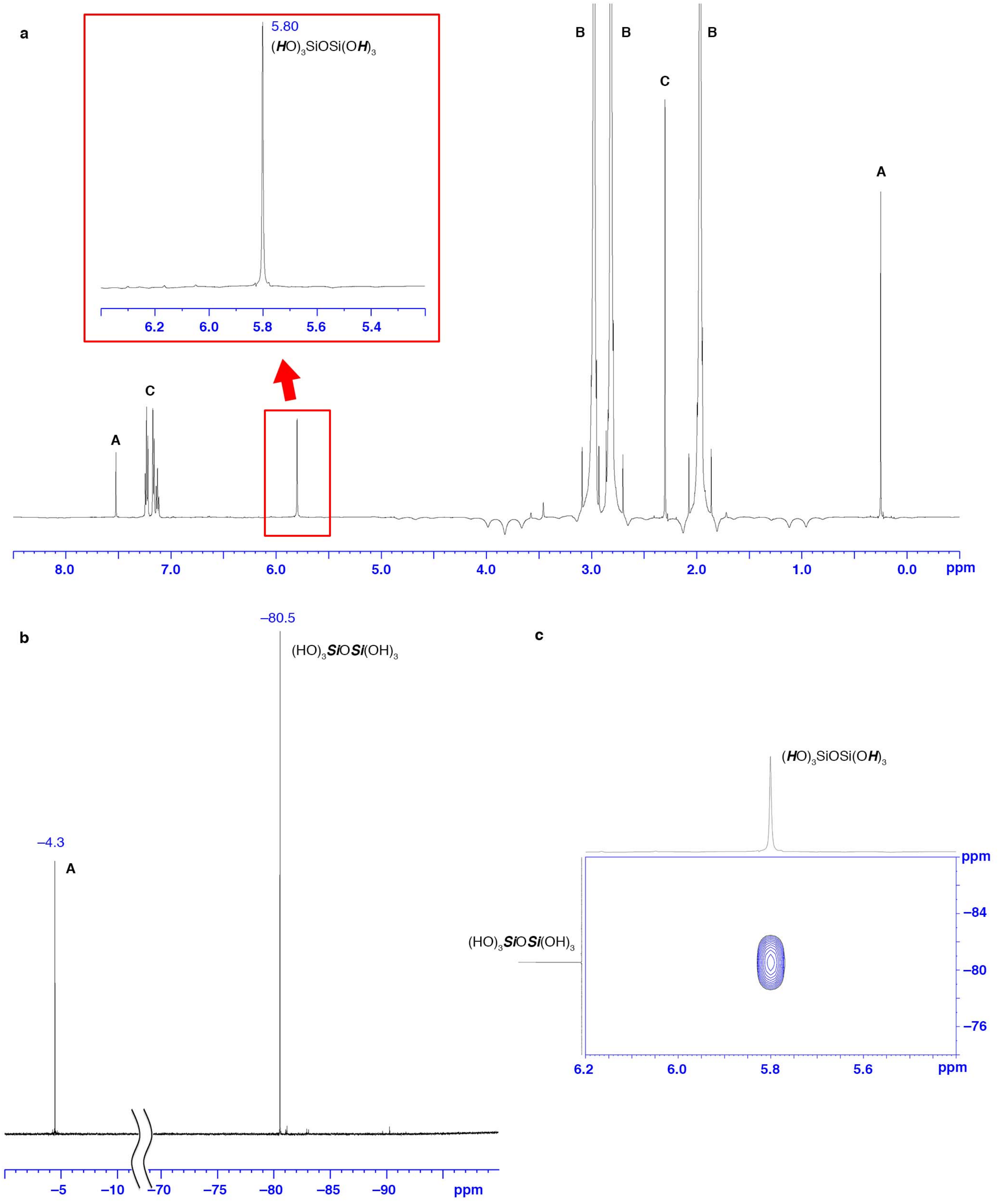

Supplementary Figure 4 | NMR spectra of the reaction mixture of the hydrogenolysis reaction of 8 in DMAc. (**a**) ^{1}H NMR spectrum. A sharp O*H* signal of disilicic acid **2** is clearly observed. Other signals: **A** belong to the standard compound 1,4-{$(CH_3)_3Si$}$_2C_6H_4$; **B** belong to the solvent (DMAc, $CH_3CON(CH_3)_2$); **C** belong to toluene, which is the co-product of the hydrogenolysis reaction. (**b**) ^{29}Si NMR spectrum. A signal of disilicic acid **2** is clearly observed. **A** is the signal of the standard compound 1,4-{$(CH_3)_3Si$}$_2C_6H_4$. (**c**) ^{1}H-^{29}Si heteronuclear multiple-bond correlation (HMBC) NMR spectrum. A cross peak is clearly observed between ^{1}H and ^{29}Si signals of disilicic acid **2**. Details of the sample preparation are shown in the Supplementary Information.

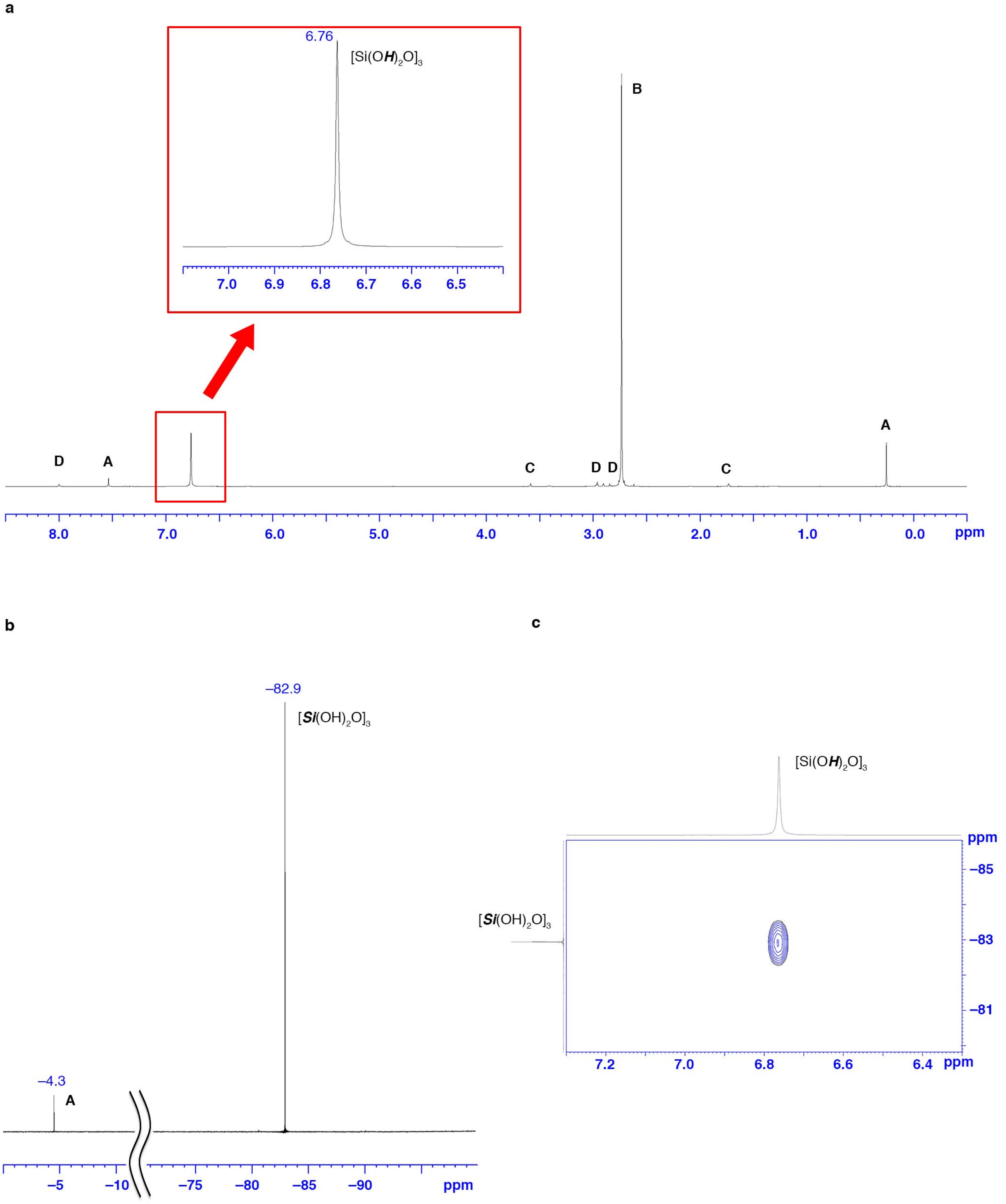

Supplementary Figure 5 | NMR spectra of the crystalline 4 containing TMU in THF-*d*$_8$ and DMF-*d*$_7$ (25:75 volume ratio). (**a**) ^{1}H NMR spectrum. Signal of **4** is clearly observed. Other signals: **A** belong to the standard compound 1,4-$\{(CH_3)_3Si\}_2C_6H_4$; **B** belongs to TMU; **C** belong to residual THF in THF-*d*$_8$; **D** belong to residual DMF in DMF-*d*$_7$. (**b**) ^{29}Si NMR spectrum. A signal of **4** is clearly observed. **A** is the signal of the standard compound 1,4-$\{(CH_3)_3Si\}_2C_6H_4$. (**c**) ^{1}H-^{29}Si heteronuclear multiple-bond correlation (HMBC) NMR spectrum. A cross peak is clearly observed between ^{1}H and ^{29}Si signals of **4**. Details of the sample preparation are shown in the Supplementary Information.

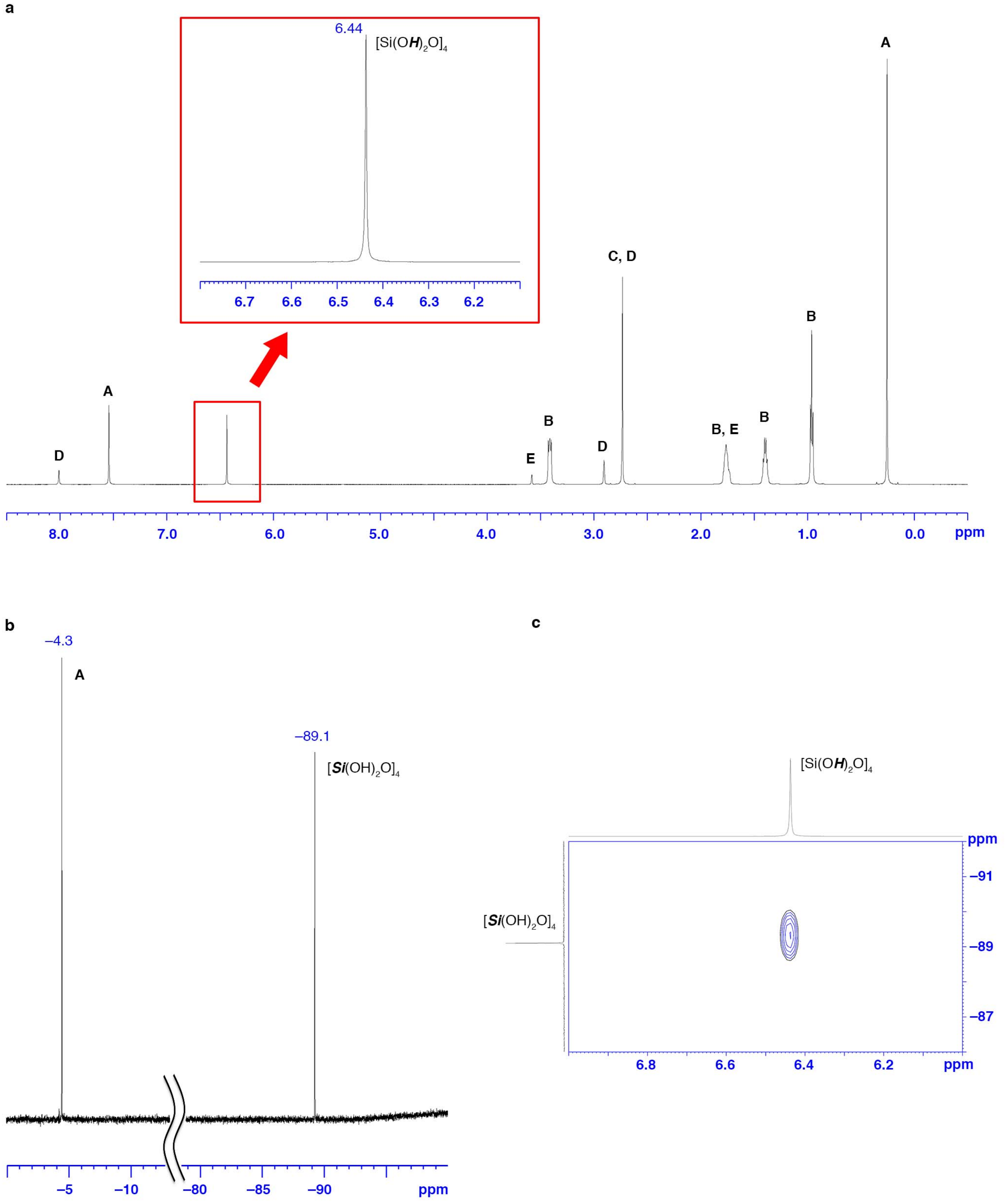

Supplementary Figure 6 | NMR spectra of the crystalline [[Si(OH)$_2$O]$_4$·4(^{n}Bu$_4$NCl)·2TMU]. (**a**) ^{1}H NMR spectrum. A sharp O*H* signal of **6** is clearly observed. Other signals: **A** belong to the standard compound 1,4-{(CH$_3$)$_3$Si}$_2$C$_6$H$_4$; **B** belong to ^{n}Bu$_4$NCl; **C** belongs to TMU, **D** belong to residual DMF in DMF-d_7; **E** belong to residual THF in THF-d_8. (**b**) ^{29}Si NMR spectrum. A signal of **6** is clearly observed. **A** is the signal of the standard compound 1,4-{(CH$_3$)$_3$Si}$_2$C$_6$H$_4$. (**c**) ^{1}H-^{29}Si heteronuclear multiple-bond correlation (HMBC) NMR spectrum. A cross peak is clearly observed between ^{1}H and ^{29}Si signals of **6**. Details of the sample preparation are shown in the Supplementary Information.

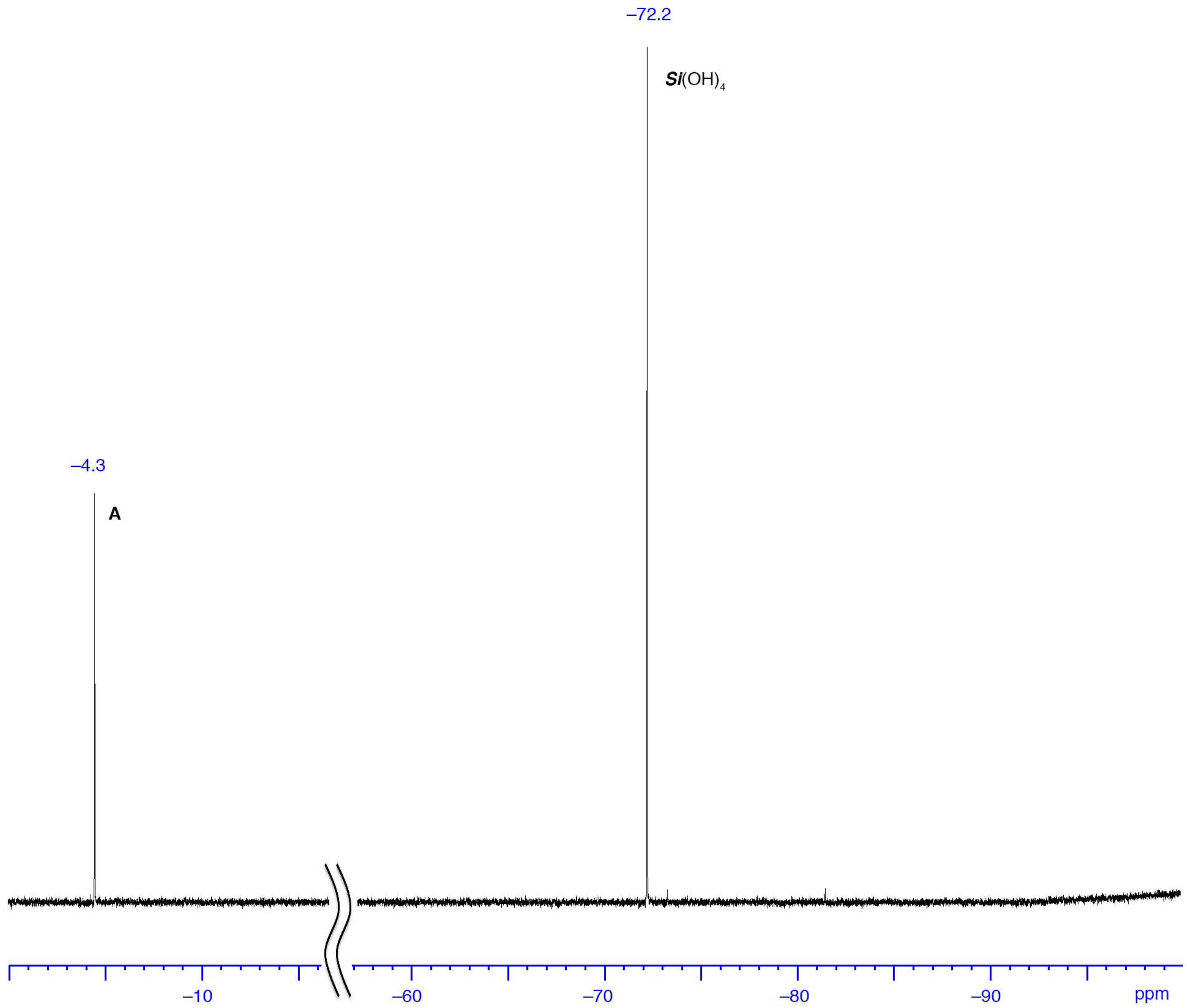

Supplementary Figure 7 | ^{29}Si NMR spectrum of orthosilicic acid obtained by the hydrolysis of Si(OCH$_3$)$_4$ in DMAc. The signal at –4.3 ppm (**A**) belong to the standard compound 1,4-{(CH$_3$)$_3$Si}$_2$C$_6$H$_4$. Details of the sample preparation are shown in the Supplementary Information.

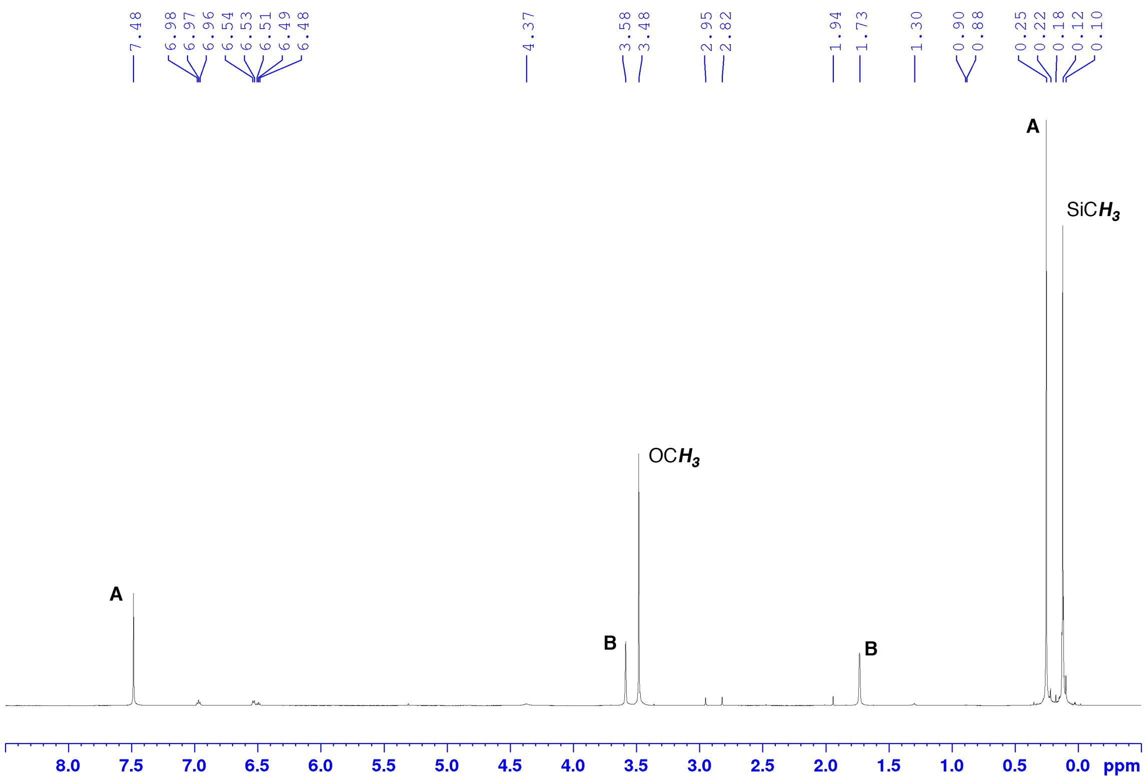

Supplementary Figure 8 | ^{1}H NMR spectra of compound 10b. Signals other than **10b**: **A** belong to the standard compound 1,4-$\{(CH_3)_3Si\}_2C_6H_4$; **B** belong to residual THF in THF-d_8.

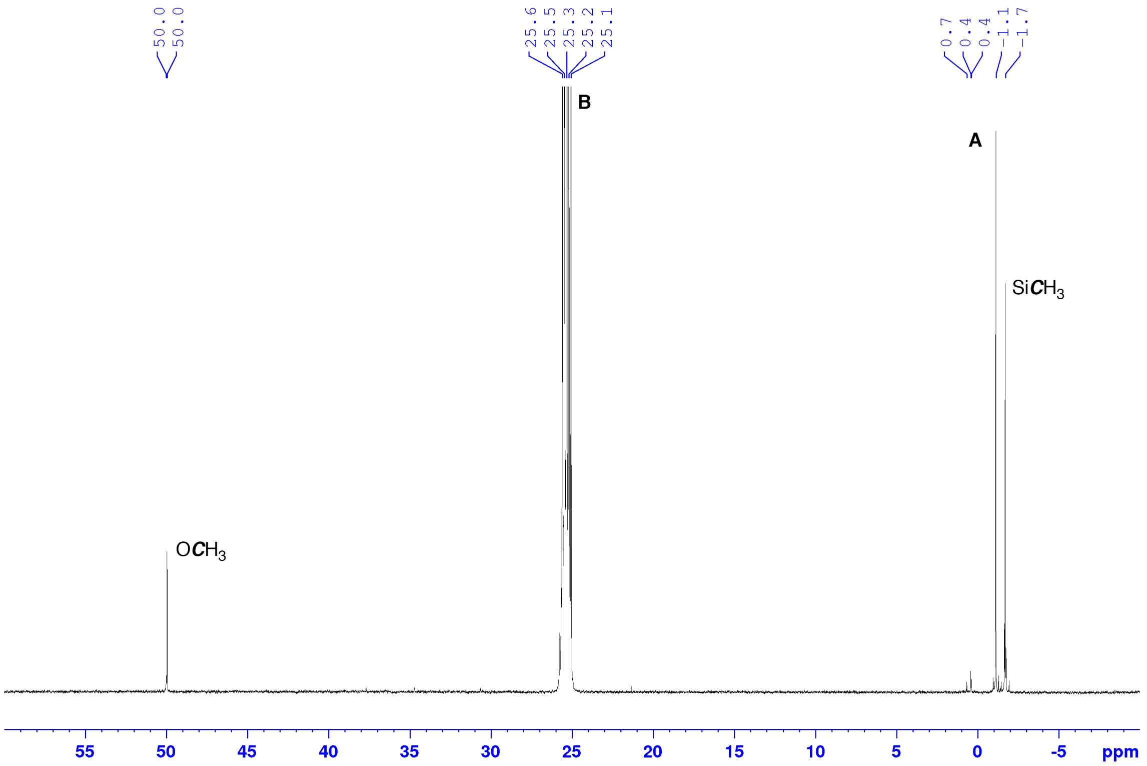

Supplementary Figure 9 | ^{13}C NMR spectra of compound 10b. Signals other than **10b**: **A** belongs to the standard compound 1,4-$\{(CH_3)_3Si\}_2C_6H_4$; **B** belongs to THF-d_8.

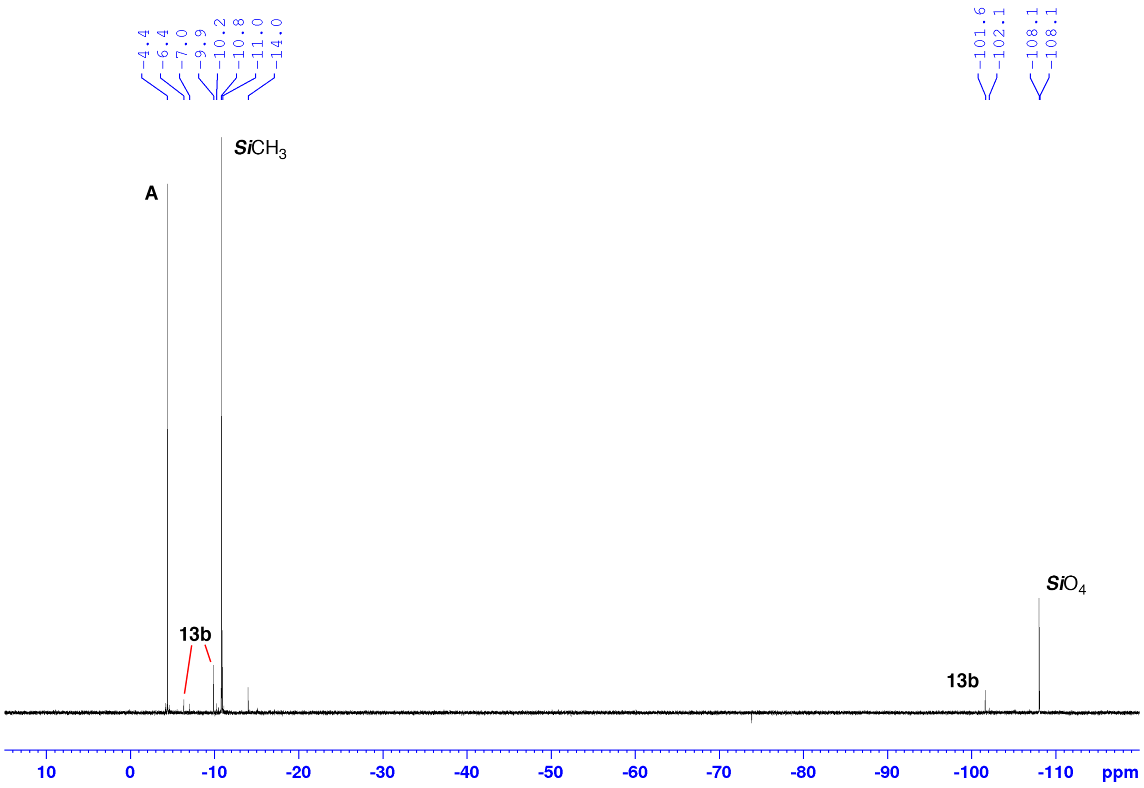

Supplementary Figure 10 | ^{29}Si NMR spectra of compound 10b. Small signals of compound **13b** are also observed. Signals other than **10b**: **A** belongs to the standard compound 1,4-$\{(CH_3)_3Si\}_2C_6H_4$.

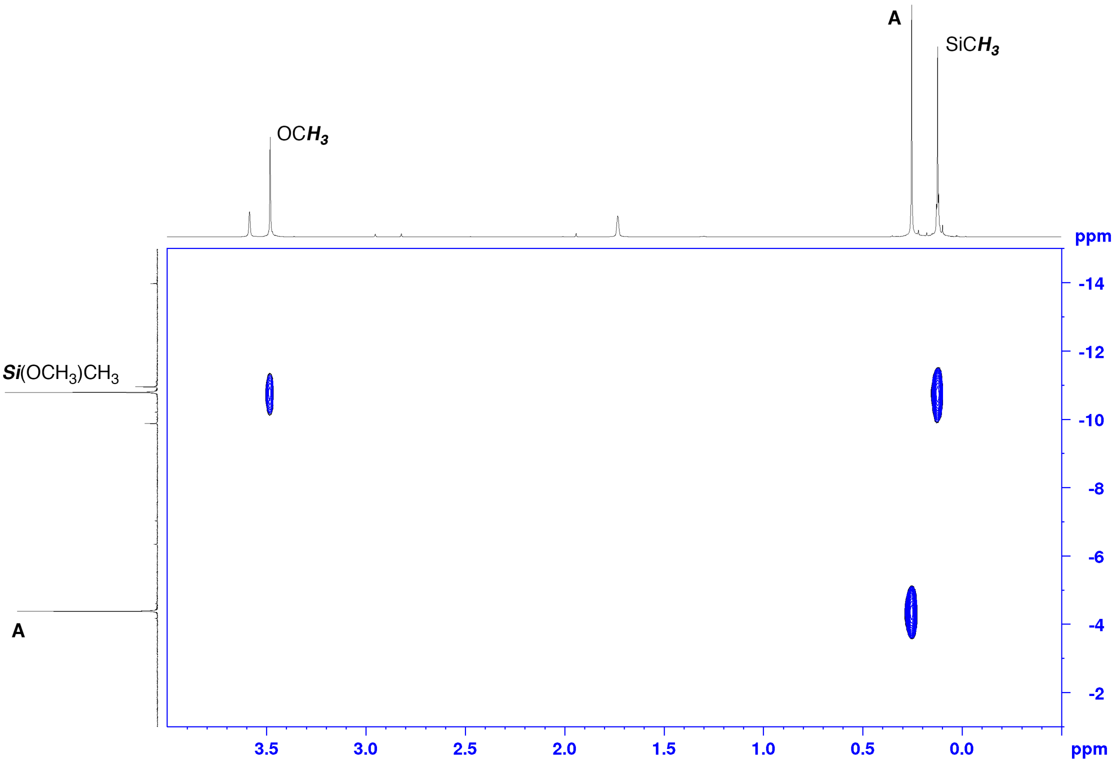

Supplementary Figure 11 | ^{1}H-^{29}Si heteronuclear multiple-bond correlation (HMBC) NMR spectrum of compound 10b. A cross peak is clearly observed between ^{1}H and ^{29}Si signals of **10b**. Signals other than **10b**: **A** belong to the standard compound 1,4-$\{(CH_3)_3Si\}_2C_6H_4$.

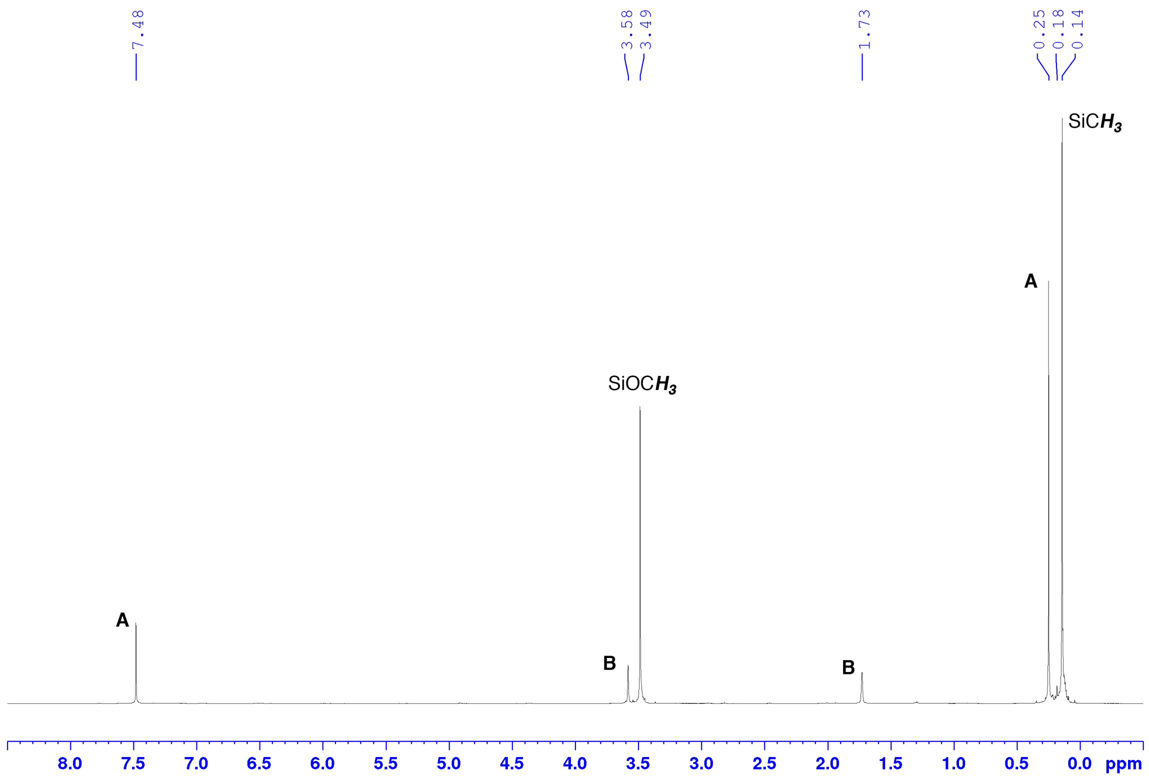

Supplementary Figure 12 | ¹H NMR spectra of compound 11b. Signals other than **11b**: **A** belong to the standard compound 1,4-$\{(CH_3)_3Si\}_2C_6H_4$; **B** belong to residual THF in THF-d_8.

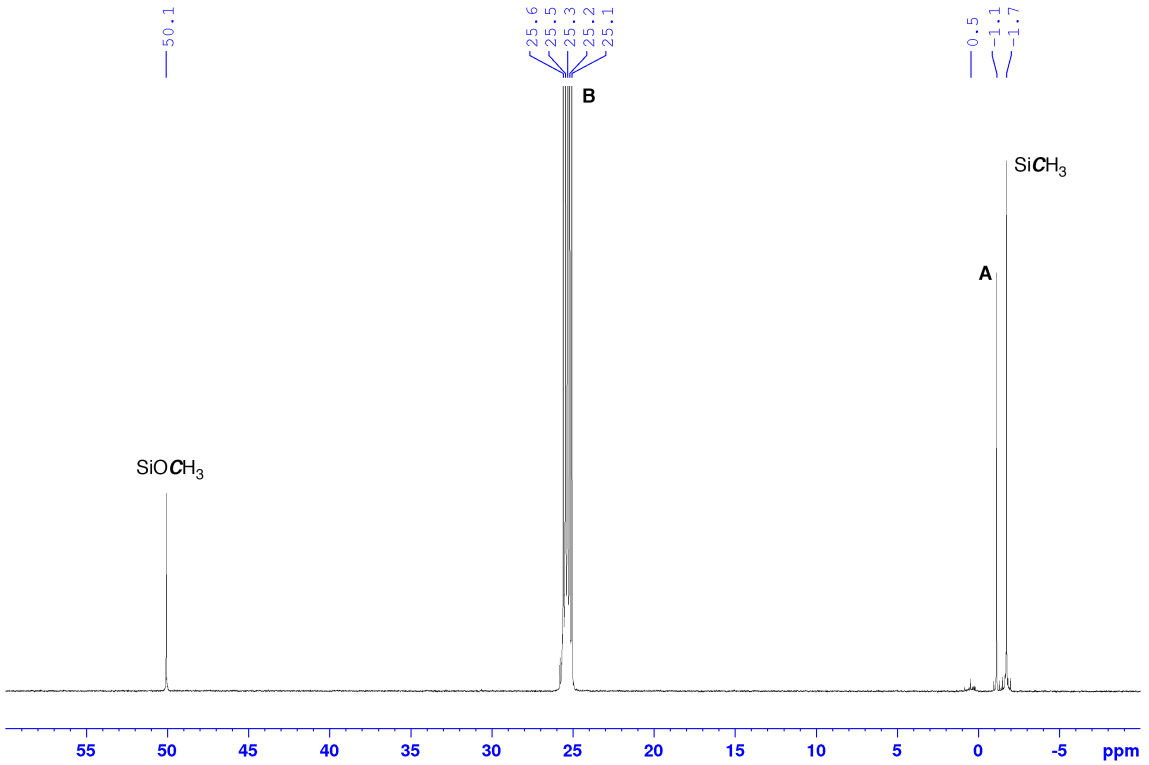

Supplementary Figure 13 | ¹³C NMR spectra of compound 11b. Signals other than **11b**: **A** belongs to the standard compound 1,4-$\{(CH_3)_3Si\}_2C_6H_4$; **B** belongs to THF-d_8.

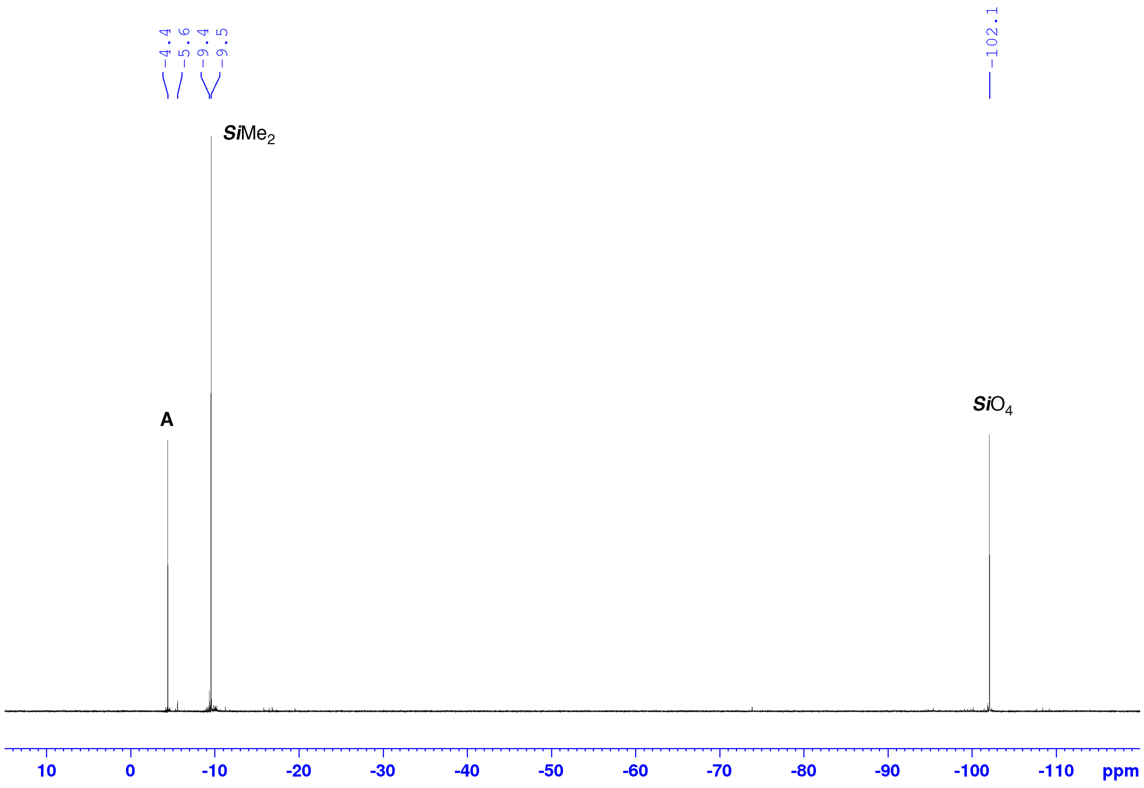

Supplementary Figure 14 | ^{29}Si NMR spectra of compound 11b. Signals other than **11b**: **A** belongs to the standard compound 1,4-$\{(CH_3)_3Si\}_2C_6H_4$.

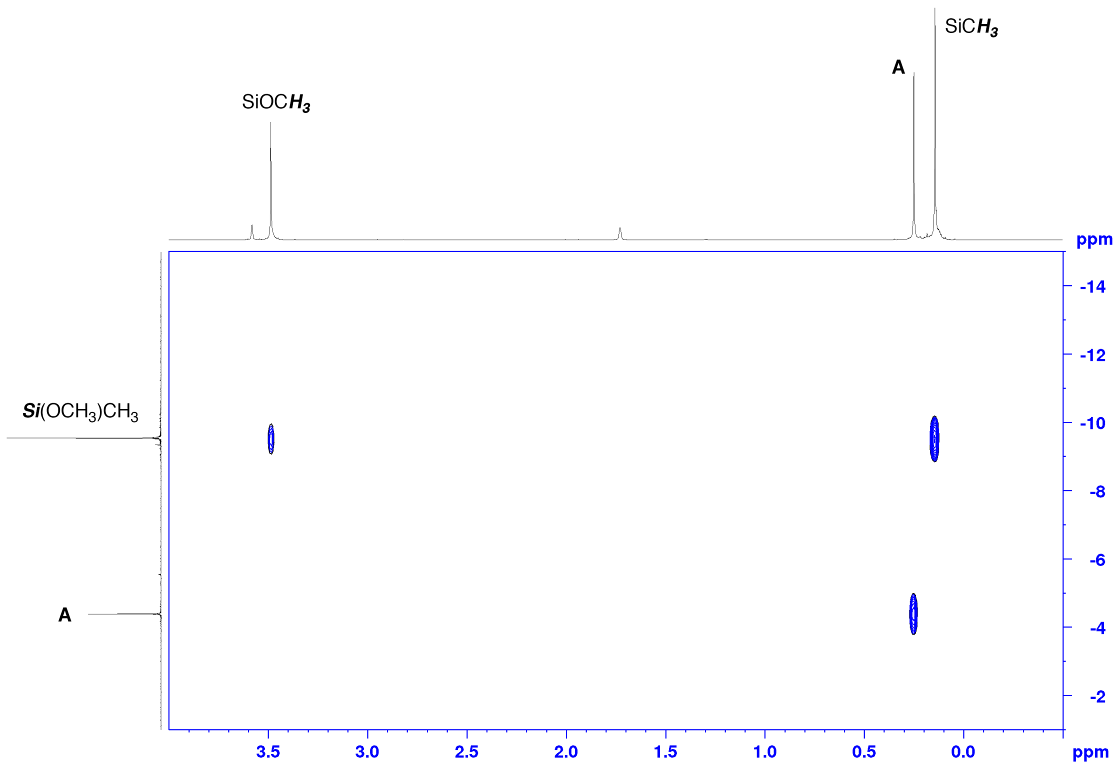

Supplementary Figure 15 | ^{1}H-^{29}Si heteronuclear multiple-bond correlation (HMBC) NMR spectrum of compound 11b. A cross peak is clearly observed between ^{1}H and ^{29}Si signals of **11b**. Signals other than **11b**: **A** belong to the standard compound 1,4-$\{(CH_3)_3Si\}_2C_6H_4$.

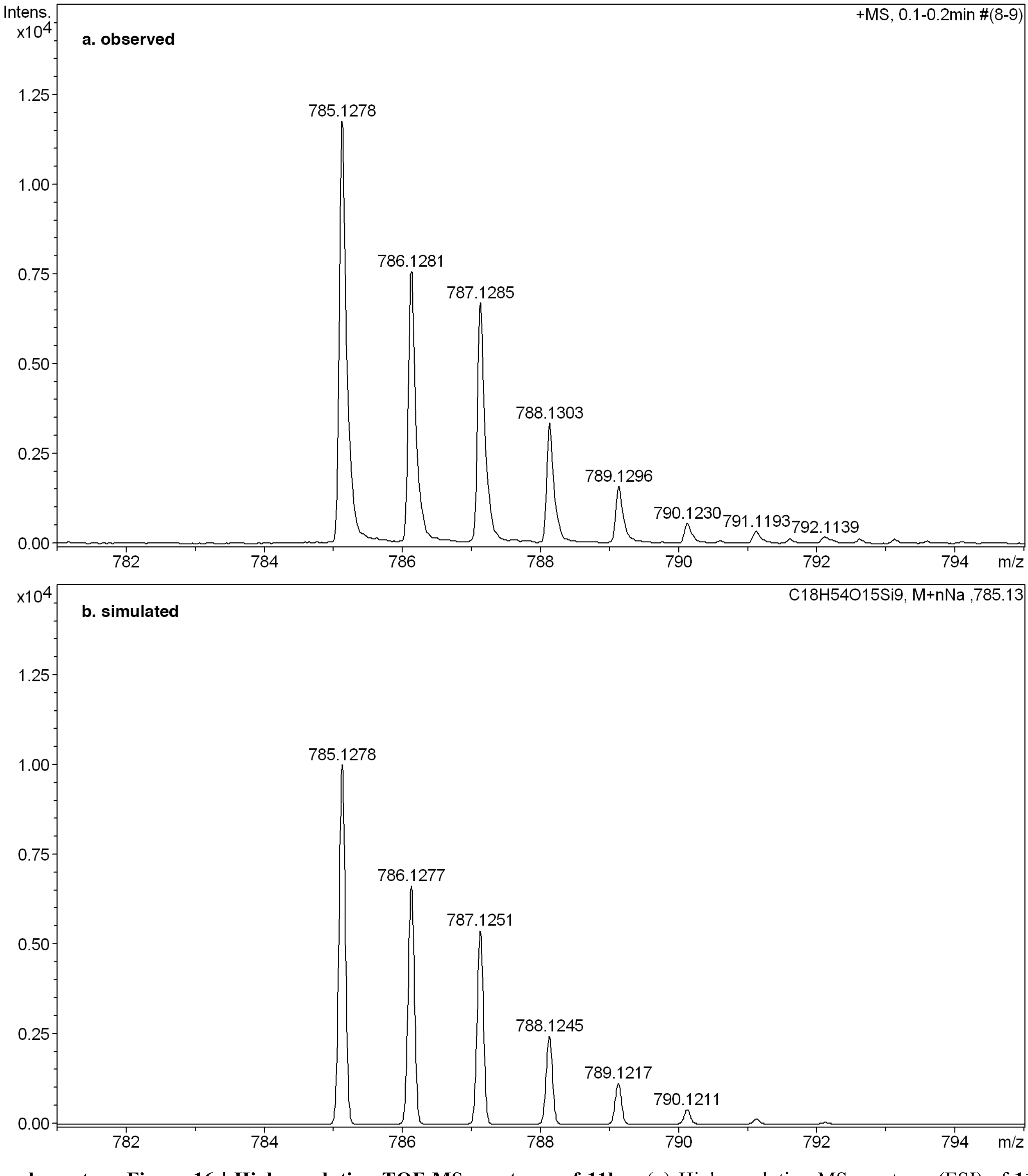

Supplementary Figure 16 | High-resolution TOF-MS spectrum of 11b. (**a**) High-resolution MS spectrum (ESI) of **11b** obtained in the positive ion mode clearly showing [**11b**+Na]$^+$ signal. (**b**) A simulated isotope pattern for [**11b**+Na]$^+$. The observed spectrum is in good agreement with the simulated spectrum.

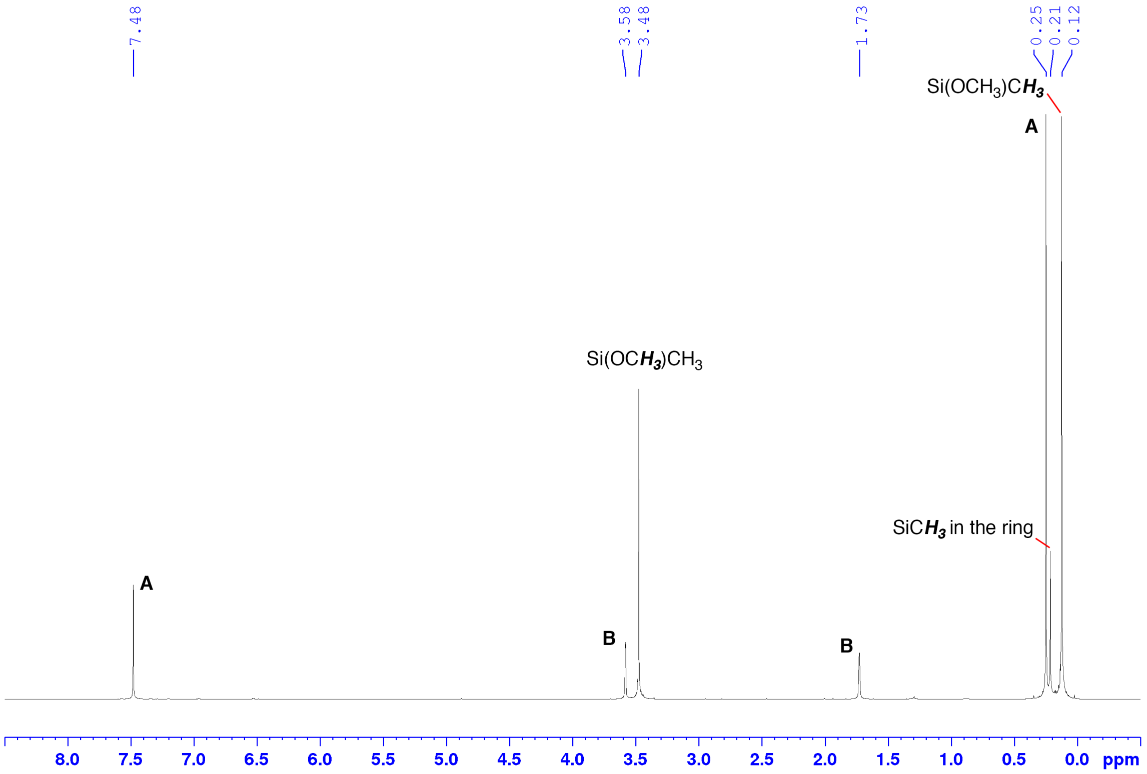

Supplementary Figure 17 | ^{1}H NMR spectra of compound 13b. Signals other than **13b**: **A** belong to the standard compound 1,4-$\{(CH_3)_3Si\}_2C_6H_4$; **B** belong to residual THF in THF-d_8.

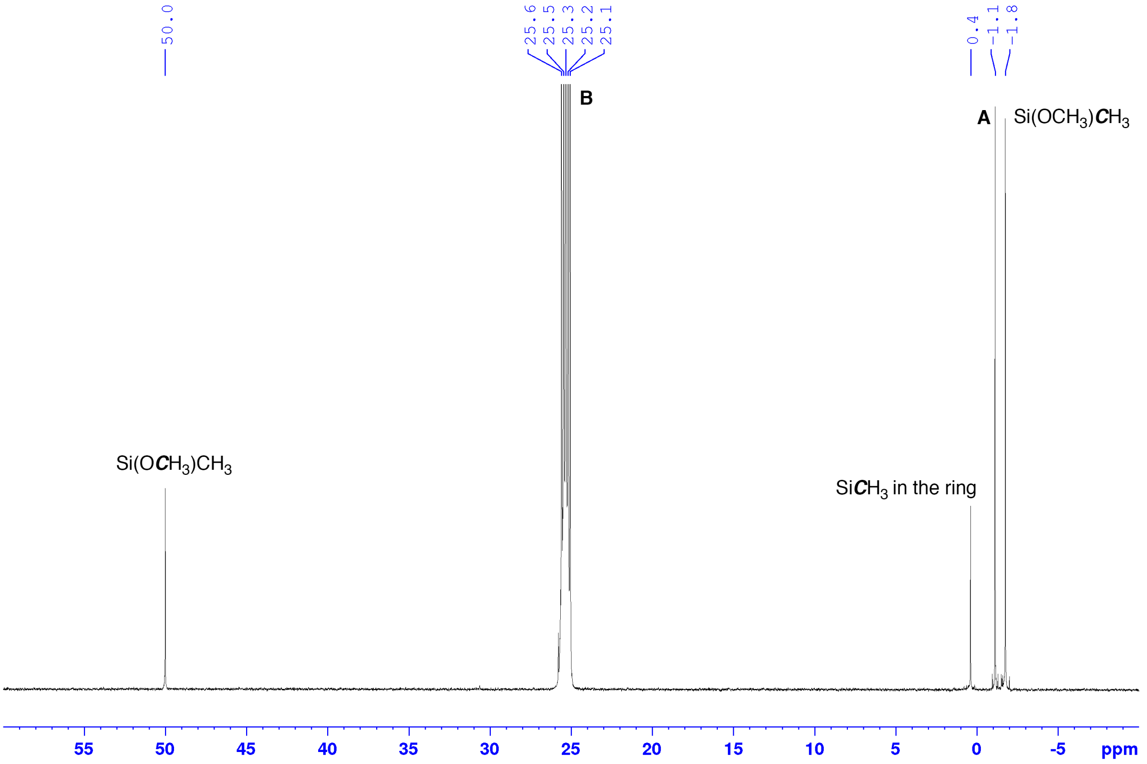

Supplementary Figure 18 | ^{13}C NMR spectra of compound 13b. Signals other than **13b**: **A** belongs to the standard compound 1,4-$\{(CH_3)_3Si\}_2C_6H_4$; **B** belongs to THF-d_8.

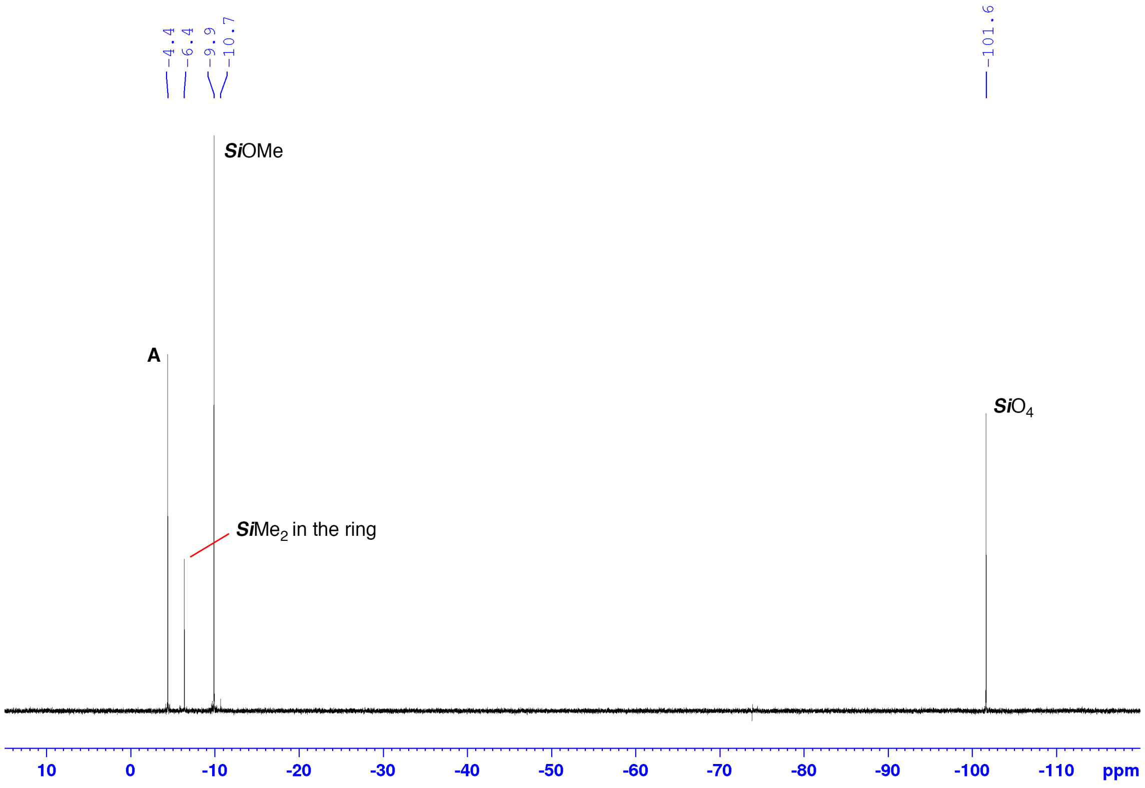

Supplementary Figure 19 | ^{29}Si NMR spectra of compound 13b. Signals other than **13b**: **A** belongs to the standard compound 1,4-$\{(CH_3)_3Si\}_2C_6H_4$.

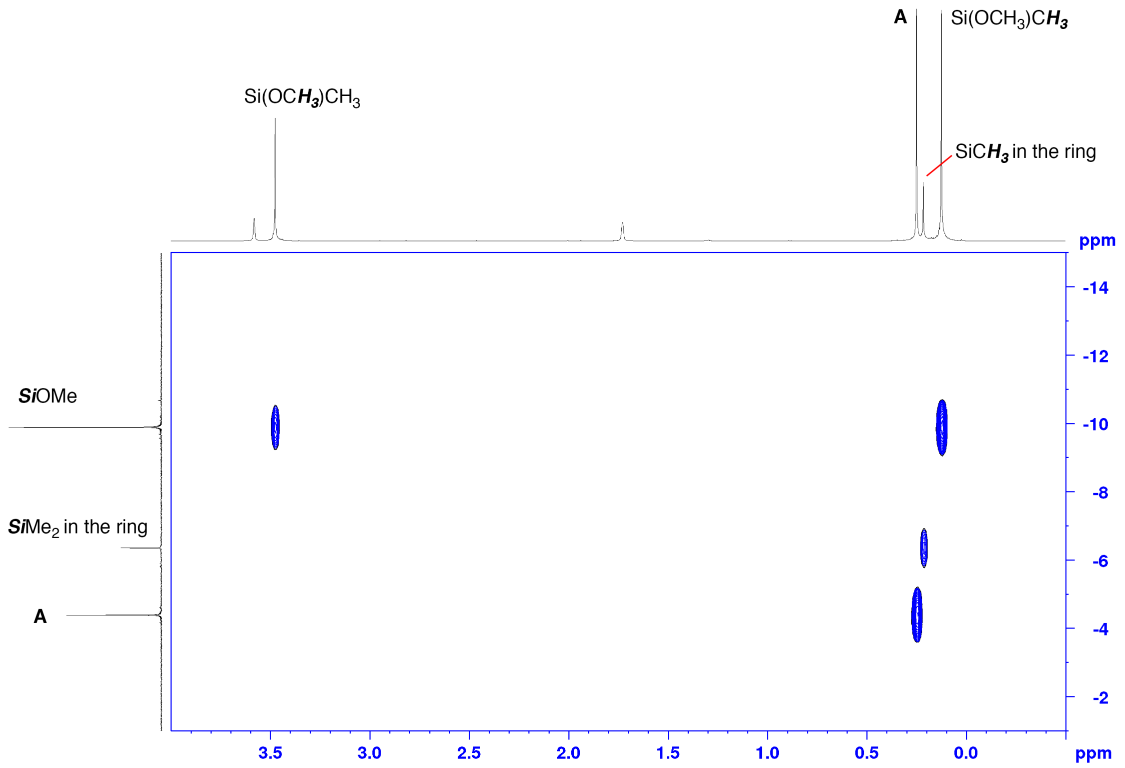

Supplementary Figure 20 | ^{1}H-^{29}Si heteronuclear multiple-bond correlation (HMBC) NMR spectrum of compound 13b. A cross peak is clearly observed between ^{1}H and ^{29}Si signals of **13b**. Signals other than **13b**: **A** belong to the standard compound 1,4-$\{(CH_3)_3Si\}_2C_6H_4$.

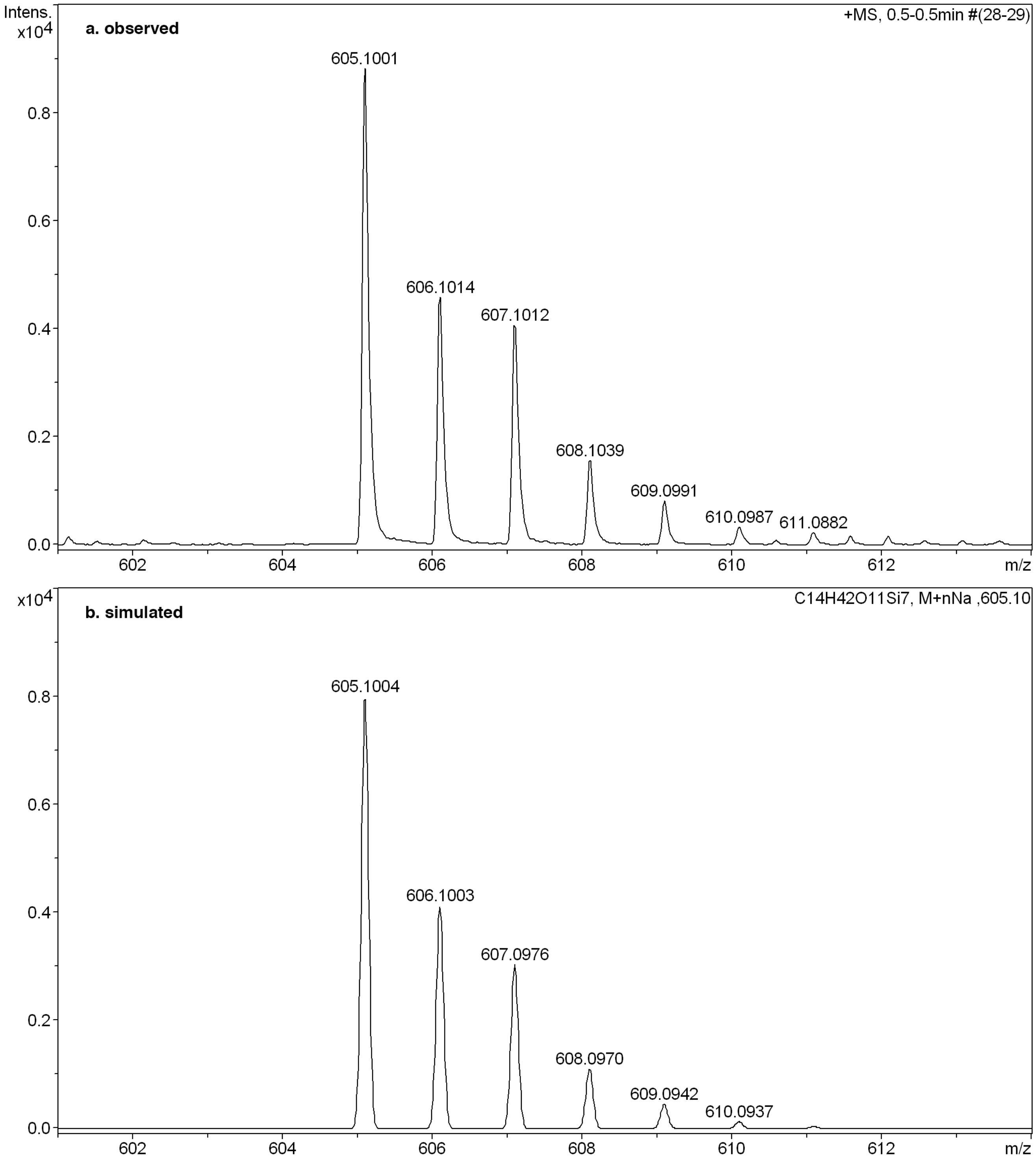

Supplementary Figure 21 | High-resolution MS spectrum of 13b. (**a**) High-resolution MS spectrum (ESI) of **13b** obtained in the positive ion mode clearly showing [**13b**+Na]$^+$ signal. (**b**) A simulated isotope pattern for [**13b**+Na]$^+$. The observed spectrum is in good agreement with the simulated spectrum.

Supplementary References

1 Chaudhary, S. K. & Hernandez, O. 4-dimethylaminopyridine - efficient and selective catalyst for the silylation of alcohols. *Tetrahedron Lett.* 99-102 (1979).

2 Beurskens, P. T. *et al.* The DIRDIF 1991 program package. *Z. Kristallogr.* Suppl. 4, 99 (1991).

3 Burla, M. C. *et al.* SIR2011: a new package for crystal structure determination and refinement. *J. Appl. Crystallogr.* **45**, 357-361 (2012).

4 Sheldrick, G. M. A short history of shelx. *Acta Crystallogr., Sect. A* **64**, 112-122 (2008).

5 Ohhara, T. *et al*. SENJU: a new time-of-flight single-crystal neutron diffractometer at J-PARC. *J. Appl. Crystallogr.* **49**, 120-127 (2016)

6 Ohhara, T. *et al.* Development of data processing software for a new TOF single crystal neutron diffractometer at J-PARC. *Nucl. Instrum. Methods Phys. Res., Sect. A* **600**, 195-197 (2009).

7 Petricek, V., Dusek, M. & Palatinus, L. Crystallographic computing system JANA2006: General features. *Z. Kristallogr.* **229**, 345-352 (2014).

8 Dolomanov, O. V., Bourhis, L. J., Gildea, R. J., Howard, J. A. K. & Puschmann, H. OLEX2: A complete structure solution, refinement and analysis program. *J. Appl. Crystallogr.* **42**, 339-341 (2009).
