# Supplementary material for: Non-aqueous selective synthesis of orthosilicic acid and its oligomers
Source: Nat Commun. 2017 Jul 26;8:140. doi: 10.1038/s41467-017-00168-5 (PMC5529440; doi:10.1038/s41467-017-00168-5)
Supplement: Supplementary file 2 — Peer Review File [file 41467_2017_168_MOESM2_ESM.pdf]

Editorial Note:

This manuscript has been previously reviewed at another journal that is not operating a transparent peer review scheme. This document only contains reviewer comments and rebuttal letters for versions considered at Nature Communications.
